# The Usability, Feasibility, Acceptability, and Efficacy of Digital Mental Health Services in the COVID-19 Pandemic: Scoping Review, Systematic Review, and Meta-analysis

**DOI:** 10.2196/43730

**Published:** 2023-02-13

**Authors:** Shaoling Zhong, Xinhu Yang, Zihua Pan, Yu Fan, Yanan Chen, Xin Yu, Liang Zhou

**Affiliations:** 1 The Affiliated Brain Hospital of Guangzhou Medical University Guangzhou China

**Keywords:** digital medicine, COVID-19, mental health services, psychological well-being, COVID-19 pandemic

## Abstract

**Background:**

After the rapid spread of the novel SARS-CoV-2, the short-term and long-term mental health impacts of the pandemic on the public, in particular on susceptible individuals, have been reported worldwide. Although digital mental health services expand accessibility while removing many barriers to in-person therapy, their usability, feasibility, acceptability, and efficacy require continued monitoring during the initial phase of the pandemic and its aftermath.

**Objective:**

In this study, we aimed to understand what mental health services are offered, whether they are practical or acceptable, and to what extent digital mental health services are effective in response to the COVID-19 pandemic across high-income and low- and middle-income countries.

**Methods:**

This study followed the Preferred Reporting Items for Systematic Reviews and Meta-Analyses (PRISMA) guidelines and the PRISMA Extension for Scoping Reviews (PRISMA-ScR) guideline. We implemented searches in PubMed (MEDLINE), Embase, PsycINFO, and Cochrane databases for studies that were published between December 2019 and November 2021 and that involved the use of digital mental health services. Two review authors screened, assessed, and extracted studies independently. The protocol was registered on the International Prospective Register of Systematic Reviews.

**Results:**

This review identified 7506 articles through database searching. In total, 65 (0.9%) studies from 18 countries with 67,884 participants were eligible for the scoping review. Of the 65 studies, 16 (24.6%) were included in the meta-analysis. A total of 15 (23.1%) studies measured the usability; 31 (47.7%) studies evaluated the feasibility; 29 (44.6%) studies assessed the acceptability; and 51 (78.5%) studies assessed the efficacy. Web-based programs (21/65, 32.3%), videoconferencing platforms (16/65, 24.6%), smartphone apps (14/65, 21.5%), and SMS text messaging (5/65, 7.7%) were the main techniques. Psychotherapy (44/65, 67.7%) followed by psychoeducation (6/65, 9.2%) and psychological support (5/65, 7.7%) were commonly used. The results of the meta-analysis showed that digital mental health interventions were associated with a small reduction in depressive symptoms (standardized mean difference=−0.49; 95% CI −0.74 to −0.24; *P*<.001) and a moderate reduction in anxiety symptoms (standardized mean difference=−0.66; 95% CI −1.23 to −1.0; *P*=.02) significantly.

**Conclusions:**

The findings suggest that digital mental health interventions may be practical and helpful for the general population, at-risk individuals, and patients with preexisting mental disorders across high-income and middle-income countries. An expanded research agenda is needed to apply different strategies for addressing diverse psychological needs and develop integrated mental health services in the post–COVID-19 era.

**Trial Registration:**

PROSPERO CRD42022307695; https://tinyurl.com/2jcuwjym

## Introduction

### Background

As of October 5, 2022, there were 624.4 million confirmed cases of COVID-19 spread over 228 countries and territories, and COVID-19 has claimed the lives of 6.6 million individuals [1]. After the rapid spread of the novel SARS-CoV-2, known risk factors for mental health impacts have been reported worldwide. Fear of the virus and containment strategies might challenge psychological well-being [2]. Social isolation, loneliness, unemployment, and loss of income after the incidence of COVID-19 have become common. These risk factors might result in mental health problems, such as anxiety, depression, and insomnia, particularly in susceptible populations that include patients with COVID-19, first-line health professionals, and older adults [3]. In a cross-sectional study by Lai et al in China [4], 50% (634/1257) of health care workers reported experiencing depressive symptoms; 45% (560/1257) had anxiety; and 34% (427/1257) reported experiencing insomnia. Increased symptoms of mental health disorders and limited access to mental health care services and social support have also been reported in people with preexisting mental health disorders [5]. Furthermore, emerging reports suggested the possibility of the long-term effects of pandemics on psychological well-being [6,7].

Although most evidence has been on the negative mental health impacts of COVID-19, a parallel area of research interest has explored how mental health services change when exposed to such stressors [8]. Web-based digital mental health services expand accessibility while removing many barriers to in-person therapy; thus, digital mental health services might be a solution in response to the challenge. Recent efforts in implementing digital mental health services have shown promising applications based on efficacy results from randomized controlled trials (RCTs). In a systematic review [9], internet-based cognitive behavioral therapy (CBT) was linked to a higher reduction in depressive symptoms at posttreatment than treatment as usual and the waiting list. Usability, feasibility, acceptability, and efficacy results at the time of pandemics, however, require continued monitoring. Knowing what mental health care service is available, whether it is feasible and acceptable, and to what extent digital mental health services take effect is crucial to inform policy decisions, such as the delivery, implementation, and target areas for applying mental health resources during the pandemic and its aftermath.

### Objective

Against this background, the objective of this scoping review and systematic review was to qualify and quantify the usability, feasibility, acceptability, and efficacy of digital mental health interventions applied for infectious disease outbreaks in the initial phase of the pandemic. We focused on the assessment of whether each of the applied techniques was usable, feasible, acceptable, and effective. We also separately synthesized data from high-income countries and low- and middle-income countries (LMICs). As there is no standard definition of usability, feasibility, acceptability, and efficacy, we considered comprehensive and broad definitions of these terms. We defined usability as program use, user engagement, and the ease of use of the services being tested, as followed in prior research [10,11]. We measured feasibility by attrition, attendance, adherence, retention, and qualitative feedback. We defined acceptability as user satisfaction, intent to continue use, and the perceived appropriateness of the intervention [12]. We defined efficacy as the intended effects and effect size estimation of the services [12].

## Methods

### Overview

This report includes a scoping review of studies on digital mental health interventions applied for infectious disease outbreaks and a systematic review of studies assessing commonly reported mental distresses identified in the scoping review. We followed the Preferred Reporting Items for Systematic Reviews and Meta-Analyses (PRISMA) guidelines and the PRISMA Extension for Scoping Reviews (PRISMA-ScR) guideline when appropriate (Multimedia Appendix 1).

### Data Search

We sought to include original studies focusing on digital mental health services applied during or in response to COVID-19. We implemented searches in PubMed (MEDLINE), Embase, PsycINFO, and Cochrane between December 2019 and November 2021 using a combination of search terms relating to both digital mental health services and COVID-19. The complete search strategy is provided in Multimedia Appendix 2. We performed a manual search of the reference lists of relevant reviews, eligible studies, and relevant conference abstracts.

### Study Selection

We removed duplicate citations across electronic databases before the screening. Initially, pairs of 2 review authors (SZ, XY, ZP, and YF) independently screened the titles and abstracts for eligibility. We referred to full texts if titles and abstracts did not provide information for eligibility based on the inclusion and exclusion criteria. The full texts of the potentially included studies were screened by 1 review author and checked by another author for agreement. In case of disagreements, the opinion of a third reviewer was sought, and a consensus was reached through discussion.

We included qualitative, quantitative, and mixed methods studies that involved the use of digital mental health services. Eligible interventions included health education, evaluation, consulting, helplines, psychological interventions, and telemedicine. The intervention was delivered by any means of digital technology, including SMS text messaging, smartphones, websites, social media, videoconferencing, wearable devices, or mobile apps. We excluded comments, protocol papers, systematic reviews, preprints, conference papers, and studies that did not provide any qualitative or quantitative data for defined outcomes. We excluded studies that were conducted before December 2019. There was no exclusion for age, gender, ethnicity, socioeconomic status, professions (health workers or not), or any type of mental disorder (clinical or nonclinical). For the meta-analysis, we included parallel RCTs and crossover RCTs that provided adequate data for analyses, using the same aforementioned criteria.

### Data Extraction

We developed a data extraction form that could be used by all review authors, as recommended by the Joanna Briggs Institute Scoping Review methodological guidance [13]. The data extraction form included information on study characteristics (authors, publication year, study design, study period, geographic regions, and study aim), participant characteristics (age, gender, sample size, and type of mental illness), digital mental health services characteristics (types of techniques and type of mental health services), and outcome characteristics (type of outcomes and their measurements). Owing to the diverse outcomes identified across eligible studies, we extracted the study results in 2 phases. In phase 1, we extracted the key findings for all reported outcomes. For effectiveness data, we extracted the results for significant differences between digital mental health services and comparison groups or trends between postintervention and preintervention outcome measurements for digital mental health services. In phase 2, we identified RCTs that reported similar digital mental health services and outcomes for the systematic review and meta-analysis. Here, effectiveness data from the comparison groups and intervention arms were retrieved using separate extraction forms. The data were extracted by 2 reviewers (XY and ZP) independently. Disagreements were resolved by discussion. A third reviewer (SZ) was involved when necessary.

### Risk of Bias Assessment

Two reviewers (XY and ZP) independently assessed the risk of bias using the Cochrane Risk of Bias (RoB 2) tool for all included RCTs. Disagreements were resolved by discussions. In case of uncertainties, a third reviewer (SZ) was consulted. The 5 domains included bias owing to the assignment of intervention, adherence to the intervention, missing outcome data, outcome measurement, and selection of reported outcome that were measured. Studies were rated as “low” risk, “some concerns,” or “high” risk.

### Data Synthesis

Owing to the nature of the research question and the heterogeneity of the studies, we synthesized qualitative and quantitative results separately. For the scoping review, we descriptively summarized the included studies with a narrative synthesis of individual studies. We summarized the overall number of included studies, the total number of study populations and their basic characteristics, countries, study design, type of digital mental health services, and the objectives and findings of the included studies. We clustered the studies by the type of techniques used and location (high-income countries vs LMICs). For each type of technique, the findings on usability, feasibility, acceptability, and effectiveness data were narratively synthesized by a textual approach.

For meta-analyses, we synthesized findings on similar efficacy outcomes that were measured by >3 RCTs. Outcomes, such as loneliness, burnout, or psychological well-being, that were measured by 1 or 2 RCTs were not synthesized. We pooled continuous outcomes as standardized mean differences (SMD) with 95% of CIs using a random-effects meta-analysis. The values of 0.2, 0.5, and 0.8 in SMDs denote minor, medium, and large effects, respectively [14]. Cochran *Q* and the *I*^2^ statistic were used to assess heterogeneity. An *I*^2^ value of ≥60% can be considered a substantial level of inconsistency across studies [15]. The analyses were stratified according to the reported outcomes. For each of these, we pooled the evidence from all eligible RCTs, regardless of the techniques applied, measurements used, and population studied. We conducted subgroup analyses to examine whether there were differences in outcomes on the basis of sample size (as a dichotomous variable), measurement of the outcome, location, duration of follow-up, and duration of intervention. We also performed meta-regression analyses to explore the effect size by sample size (as a continuous variable), age, and proportion of females in the population studied. We used funnel plots and ran the Egger test to look for asymmetry to detect publication bias that might impair the validity of our findings. We performed all analyses with R statistical software (version 4.2.2; R Foundation for Statistical Computing) [16].

## Results

### Study Selection

**Figure 1:**
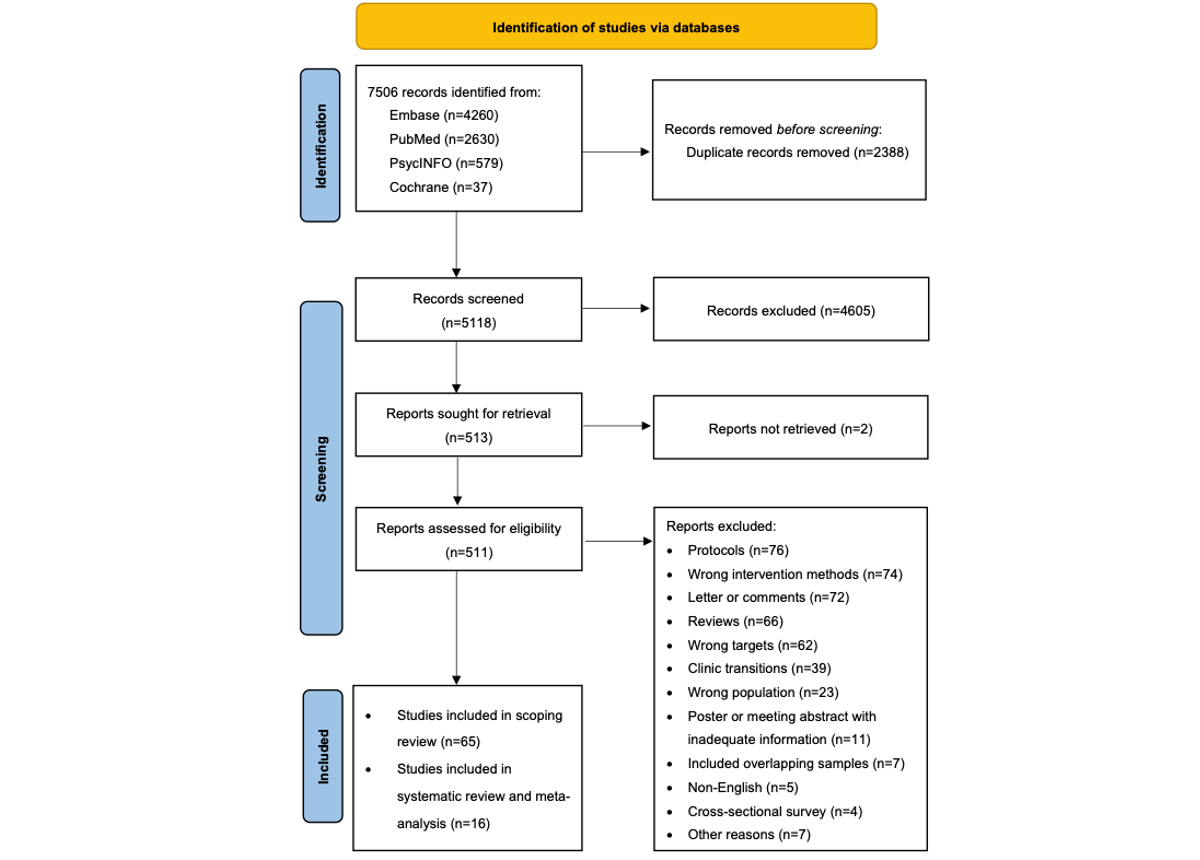
Flowchart of study selection.

The PRISMA flowchart shows the study selection process (Figure 1). This review identified 7506 articles that were identified through database searching. After removing duplicate records, we screened 5118 (68.19%) records and identified 65 (1.27%) eligible studies for the scoping review, and the results were synthesized narratively. Of the 19 RCTs, 17 (89%) studies reported depressive symptoms and 18 (95%) studies reported anxiety symptoms as the outcome. In total, 3 (16%) studies were not included in the meta-analyses; 2 (67%) studies [17,18] that reported on depression and anxiety disorders did not provide adequate data, and 1 (33%) study that reported anxiety scores using state anxiety was with a very large estimate [19]. Accordingly, of the total 65 eligible studies, 16 (25%) studies that reported depressive symptoms or anxiety symptoms were included in the meta-analysis.

### Study Characteristics

Table 1 presents the characteristics of participants and studies included in the review. The 65 included studies were conducted in the following 18 different countries: 24 (37%) studies from the United States (participants: 54,245/67,884, 79.9%), 8 (12%) studies from China (1114/67,884, 1.6%), 6 (9%) studies from Canada (5490/67,884, 8.1%), 5 (8%) studies from Italy (315/67,884, 0.5%), 3 (5%) studies from Australia (1004/67,884, 1.5%), 3 (5%) studies from France (2235/67,884, 3.3%), 2 (3%) studies from India (654/67,884, 1%), 2 (3%) studies from Malaysia (123/67,884, 0.2%), 2 (3%) studies from Greece (164/67,884, 0.2%), 2 (3%) studies from Turkey (121/67,884, 0.2%), and further 9 (14%) studies were conducted in other countries (ie, Dominican Republic, Israel, Japan, Oman, South Africa, Spain, Sweden, and the United Kingdom). Of these, 49 (75%) studies were from high-income countries, and 16 (25%) studies were conducted in middle-income countries. None of the included studies reported data from low-income countries. The participant pool consisted of 67,884 participants; most (44,125/67,884, 65%) were female. There were 2663 (3.9%) people with a mental illness or mental distress, 713 (1.1%) people with or suspected of COVID-19, and 748 (1.1%) hospital workers.

Of the 65 studies, a total of 15 (23.1%) studies measured usability; 31 (47.7%) evaluated feasibility; 29 (44.6%) assessed acceptability; and 51 (78.5%) assessed efficacy.

Web-based programs (21/65, 32.3%), videoconferencing platforms (16/65, 24.6%), smartphone apps (14/65, 21.5%), and SMS text messaging (5/65, 7.7%) were the main techniques applied. There were other studies that used social media (3/65, 4.6%), hotlines (3/65, 4.6%), a chatbot (1/65, 1.5%), virtual reality (VR; 1/65, 1.5%), and robotic telemedicine (1/65, 1.5%). Most studies provided psychotherapy (44/65, 67.7%), followed by psychoeducation (6/65, 9.2%), psychological support (5/65, 7.7%), psychological assessments (4, 6.1%), and psychiatric clinical services (4/65, 6.1%).

**Table 1:** Characteristics of participants and studies included in the qualitative systematic review.

Author and year	Design and study type	Sample size, n	Age (years), mean (SD)^a^	Female, %	Intervention vs control group
Aguilera et al [20]	Pre- and postintervention evaluation; quantitative	303	33.3 (11.0)	78	A texting intervention (StayWell at Home)
Agyapong et al [21]	Pre- and postintervention evaluation; quantitative	2767	76.7% were aged between 26 and 60	88	Text4Hope subscribers who received once-daily supportive SMS text messages vs Text4Hope subscribers who did not receive messages
Al-Alawi et al [18]	RCT^b^; quantitative	46	28.51 (8.7)	78	Therapist-guided web-based therapy vs newsletter received via email containing self-help information and tips to cope with distress
Al-Refae et al [22]	RCT; quantitative	165	25.24 (8.74)	78.8	Smartphone intervention (Serene) vs waiting list
Bantjes et al [23]	Pre and postintervention evaluation; quantitative	175	22.2 (4.9)	85.4	A web-based group CBT^c^ intervention
Ben-Zeev et al [24]	RCT; quantitative	315	37.89 (11.64)	83.8	Smartphone app (CORE) vs waiting list
Brouzos et al [25]	Pre- and postintervention evaluation; quantitative	82	33.07 (9.55)	78	Web-based positive psychology intervention (“Staying Home—Feeling Positive”) vs no intervention
Casas [26]	Pre- and postintervention evaluation; mixed methods	15	39.52 (13.57)	73.3	Written exposure therapy delivered via telehealth
Chandra et al [27]	Pre- and postintervention evaluation; mixed methods	643	49.57 (15.23)	37.2	Mental health support telecounseling
Coifman et al [28]	Pre- and postintervention evaluation; quantitative	28	45.33 (9.6)	75	Daily coping toolkit intervention
Comer et al [29]	RCT; quantitative	40	Children=6.2 (1.8); parents=38.39 (4.5)	Children: 72.5; parents: 75	iCALM Telehealth Program vs waiting list
Craig et al [30]	Pre- and postintervention evaluation; mixed methods	96	22.3 (4.1)	17.7	AFFIRM online group vs waiting list
Detweiler Guarino et al [31]	Pre- and postintervention evaluation; quantitative	2484	44 (15.2)	53.2	The PATH program
Dincer and Inangil [32]	RCT; quantitative	72	33.46 (9.6)	88.9	Received emotional freedom techniques vs stayed comfortable in a calm and tranquil environment
Esentürk and Yarımkaya [33]	Pre- and postintervention evaluation; mixed methods	14	Children=12.07; parents=51.4	Children=42.9; parents=64.2	WhatsApp-based physical activity intervention
Fiol-DeRoque et al [34]	RCT; quantitative	482	41.37 (10.4)	83.2	PsyCovidApp vs a general control app
Gabrielli et al [35]	Pre- and postintervention evaluation; mixed methods	71	20.6 (2.4)	68	Healthy-coping intervention via chatbot
Geoffroy et al [36]	Postintervention evaluation;	149	32.7 (11.0)	86	Covid-Psy hotline
Gordon et al [37]	Pre- and postintervention evaluation; mixed methods	99	51.4 (14.7)	86	A guided imagery mobile app
Graziano et al [38]	Pre- and postintervention evaluation; quantitative	30	Patients=22.5 (6.9); parents=37 (6.3)	Patient=56.3; parents=92.9	Telehealth psychological interventions
Gromatsky et al [39]	Pre- and postintervention evaluation; mixed methods	20	54.2 (11.95)	15	VA CONNECT
Guan et al [19]	RCT; quantitative	95	18.63 (0.75)	28.4	Self-compassion writing induction condition vs a writing control condition (to think about a negative event)
Guo et al [40]	Pre- and postintervention evaluation; quantitative	508	Intervention=47.0 (4.3), control=46.6 (4.2)	84.1	Web-based education program vs no intervention
Held et al [41]	Pre- and postintervention evaluation; mixed methods	2	—^d^	0	Intensive cognitive processing therapy–based program
Hom et al [42]	Postintervention evaluation; mixed methods	23	39.04 (16.15)	52.2	Virtual partial hospital program
Hosseinzadeh Asl [43]	RCT; quantitative	49	33.06 (6.02)	55.1	Mindfulness-based intervention vs waiting list
Hu et al [44]	Pilot RCT; quantitative	48	42.6 (15.8)	54.2	WeChat-based psychological interventions vs conventional nursing
Ibrahim et al [45]	Pre- and postintervention evaluation; quantitative	43	63.41	67.4	Virtual group exercise
Jaworski et al [46]	Postintervention evaluation; quantitative	49,287	—	—	COVID coach
Kahlon et al [47]	RCT; quantitative	240	69.1 (12.1)	79	Receive calls vs no calls
Karagiozi et al [48]	RCT; quantitative	82	44.5 (10.6)	—	Web-based intervention group vs onsite intervention
Kim [49]	RCT; quantitative	80	41.7 (13.8)	56.25	Teleacupressure self-practice group vs web-based communication group
Kolbe et al [50]	Postintervention evaluation; mixed methods	13 patients and 11 staff	—	—	Virtual reality
Lazzaroni et al [51]	Pre- and postintervention evaluation; quantitative	50	Between 13 and 24	84	Eye movement desensitization and reprocessing intervention group
Lima et al [52]	Postintervention evaluation; mixed methods	Healthy volunteers=10; older adults and people with dementia=1	Healthy volunteers=21-59; older adults and people with dementia=not mentioned	Healthy volunteers=50; older adults and people with dementia=0	Robotic telemedicine
Maldonado [53]	Pre- and postintervention evaluation; quantitative	40	44.1 (13)	82.5	Online intervention group
McKeon et al [54]	Pre- and postintervention evaluation; quantitative	11	64.4 (4.3)	82	Mental health–informed lifestyle intervention
Nauphal et al [55]	Pre- and postintervention evaluation; quantitative	5	36.2 (9.5)	40	Web-based CBT group
Ortiz et al [56]	Pre- and postintervention evaluation; mixed methods	383	46.9 (16.8)	70.1	Texting intervention group
Parolin et al [57]	Pre- and postintervention evaluation; mixed methods	134	33.20 (10.61)	86	Italia Ti Ascolto intervention group
Philip et al [58]	Pre- and postintervention evaluation; quantitative	2069	43.52 (13.94)	65.1	KANOPEE group
Puspitasari et al [59]	Pre- and postintervention evaluation; quantitative	76	36.6 (13.4)	86	Adult Transitions Program teletherapy group
Rojas et al [60]	Pre- and postintervention evaluation; mixed methods	1	—	0	Brief CBT for suicide prevention
Shalaby et al [61]	Postintervention evaluation; quantitative	2032	44.58 (13.45)	88	Text4Hope intervention group
Shapira et al [62]	Pilot RCT; quantitative	82	72 (5.63)	—	A short-term digital group vs a waitlist control group
Sharrock et al [63]	Pre- and postintervention evaluation; quantitative	904	37.83 (12.64)	67.1	iCBT^e^ group during COVID-19 vs iCBT group before COVID-19
Song et al [64]	Pre- and postintervention evaluation; quantitative	129	34.64 (9.11)	69	Mobile internet CBT group vs a waitlist control group
Sosa Lovera et al [65]	Postintervention evaluation; quantitative	497	32	73	COVID-19 helpline group
Sturgill et al [66]	Pre- and postintervention evaluation; quantitative	99	19.9 (1.94)	69	Ajivar activities intervention + routine mental wellness instruction group vs group that received routine mental wellness instruction
Suffoletto et al [67]	Pilot RCT; quantitative	52	18.7 (0.46)	86.5	MoST-MH^f^ Intervention vs enhanced usual care group
Summers, Wu, and Taylor [68]	Pre- and postintervention evaluation; quantitative	273	49.6 (9.24)	59.3	Gro Health app users
Sun et al [69]	Pilot RCT; mixed methods	114	22.21 (2.67)	73.7	Mindfulness-based mHealth^g^ group vs social support–based mHealth group
Tarquinio et al [70]	Pre- and postintervention evaluation; quantitative	17	33.2 (4.10)	100	Eye Movement Desensitization and Reprocessing intervention group
Vallefuoco et al [71]	Postintervention evaluation; quantitative	30	—	—	18 therapists and 12 parents of children with ASD^h^
van Agteren et al [72]	Pre- and postintervention evaluation; quantitative	89	38.67 (13.06)	66	Internet-based group mental health intervention
Wagner [73]	Postintervention evaluation; mixed methods	Children: 204; clinicians: 9	Children=27.54 months (5.36 months)	23	ASD-PEDS intervention group
Wahlund et al [74]	RCT; mixed methods	670	46 (13.50)	81.6	Web-based psychological intervention vs waiting list
Wasil et al [75]	Pre- and postintervention evaluation; quantitative	189	31.04 (8.91)	72.99	COMET intervention
Wei et al [17]	RCT; quantitative	26	44.65 (12.09)	38.5	An internet-based integrated intervention vs support care
Wood et al [76]	Postintervention evaluation; mixed methods	7	26.9 (4.8)	—	Group teletherapy
Ying et al [77]	Pre- and postintervention evaluation; quantitative	137	73.39 (7.37)	68.5	iCBT group
Zepeda et al [78]	Pre- and postintervention evaluation; mixed methods	27	9.56	67	iCOPE
Zhang et al [79]	Pilot RCT; quantitative	57	50.12 (6.79)	47.1	WeChat-based psychological interventions vs waitlist group
Zimmerman et al [80]	Postintervention evaluation; quantitative	480	37.1 (14)	67.5	Telehealth program vs partial in-person program before the COVID-19 outbreak
Van Lieshout et al [81]	RCT; quantitative	403	31.8 (4.40)	100	Web-based CBT + treatment as usual vs treatment as usual

^a^Where mean (SD) was not available in the original study, mean or ranges were used where applicable.

^b^RCT: randomized controlled trial.

^c^CBT: cognitive behavioral therapy.

^d^Not applicable.

^e^iCBT: internet-based congnitive behavioral therapy.

^f^MoST-MH: Mobile Support Tool for Mental Health.

^g^mHealth: mobile health.

^h^ASD: autism spectrum disorder.

### Qualitative Synthesis of Results

#### Overview

This section summarizes the findings from 65 studies that reported the feasibility, acceptability, and effectiveness of digital mental health services during the COVID-19 pandemic. The synthesis was organized according to the type of digital technique used. Table 2 presents a summary of usability, feasibility, acceptability, and efficacy by the types of techniques in high-income countries and LMICs. Table S1 (Multimedia Appendix 3 [17-81]) presents summaries of individual studies on mental health interventions and major findings and their measurements.

**Table 2:** Summaries of usability, feasibility, acceptability, and efficacy by type of techniques in high-income countries (HICs) and low- and middle-income countries (LMICs).

Techniques and groups			Usability		Feasibility		Acceptability		Efficacy
**Videoconferencing platforms**									
	HICs (n=11)	—^a^		Three studies reported a completion rate between 92% and 100%. Three studies reported qualitative feedback of highly perceived benefits. Three studies reported technology-related challenges.		Five studies reported high levels of overall satisfaction.		Nine studies reported psychotherapy improved depressive levels and 5 studies found improved anxiety symptoms. One study found no effects on decreasing anxiety symptoms.	
	LMICs (n=5)	—		Two studies reported the mean attendance ranges from 6.4 (SD 2.8) to 10.26 (SD 7.02).		One study reported high levels of satisfaction.		Three studies reported improvements in depressive symptoms or negative effects. Two studies reported no effects on decreasing depression levels.	
**Web-based programs**									
	HICs (n=17)	Two studies reported a significant increase in registered users. One study reported a high rating in usability.		Five studies reported the completed rate ranged from 30.5% to 85%. Three studies reported retention rate, which ranged from 30.5% to 85%. One study reported high attrition rate of 13%. One study reported strong attendance. Two studies reported that retentions were strong. Two studies reported high ratings of feasibility, and one study reported technical difficulty.		Five studies reported high satisfaction. One study reported that acceptability scores were significantly better than average. Three studies reported high acceptability and rated the interventions as acceptable, helpful, appropriate, and positive.		Eight studies reported improvement in anxiety symptoms and anxiety-related social impairment neither with nor without controls. Two studies indicated no effects on reducing anxiety. Five studies indicated improvement in depression symptoms and depression severity. One study reported no difference in depressive symptoms when compared with onsite groups. Three studies reported improved stress-related symptoms. Two studies reported reductions in COVID-19–related worry. One study indicated improvements in insomnia.	
	LMICs (n=4)	One study reported the mean time spent was 35.63 (SD 25.41) min.		One study reported a completion rate of 87.4%. One study reported high attendance and high retention rates (91.2%).		One study reported high ratings of acceptability and the other a high level of satisfaction.		Four studies indicated improvement in anxiety symptoms neither with no controls nor control groups. Two studies reported improvement in depressive symptoms with no controls. One study reported a reduction in stress and burnout.	
**Smartphone apps**									
	HICs (n=11)	Two studies reported high usability scores. Two studies reported user engagement. The time ranged from 36.7 min over 12 weeks to 1424 min over 14 weeks.		One study reported the number of days retained was 42.44 (SD 44.40). Three studies reported a retention rate that ranged from 28.3% to 85.7%.		Two studies rated the intervention as acceptable, and 2 studies rated the intervention as satisfied. One rated the intervention as good. One rated the intervention as helpful.		Four studies reported reduced depressive symptoms compared with the preintervention and waitlist groups. Five studies reported similar improvements in anxiety symptoms compared with the preintervention, waitlist group, and a control app with limited access to psychoeducational content.	
	LMICs (n=3)	—		—		One study indicated high satisfaction and would recommend the service. One study reported helpful and enjoyable.		Two studies reported improvements in depressive symptoms, insomnia, psychological flexibility, and self-compassion, either with no controls or in comparison with the waitlist group.	
**Texting**									
	HICs (n=5)	One study reported high usability.		Two studies reported a completion rate of 16% and 78%, respectively.		One study reported a high level of overall satisfaction.		Four studies reported an improved mood rating was observed at posttreatment assessment either with or without the control group.	
	LMICs (n=0)	—		—		—		—	
**Social media**									
	HICs (n=1)	—		One study reported high retention.		One study reported high acceptability.		One study reported an effect on psychological distress, quality of life, functioning, loneliness, and physical activity without a control group.	
	LMICs (n=2)	One study reported an average user time of 18.7 hours.		—		—		Two studies reported improvements in anxiety and depressive symptoms when compared with either the usual care or the waitlist group.	
**Hotline and telephone calls**									
	HICs (n=2)	One study reported 5.73 (SD 3.22) calls/day.		One study reported a dropout rate of 7.5%.		—		One study reported improvements in loneliness, depression, anxiety, and general mental health.	
	LMICs (n=1)	—		—		One study reported high satisfaction, and the participants would use again, and would recommend it to others.		—	
**Robotic telemedicine and VR^b^**									
	HICs (n=2)	One study reported an average of 78 (SD 24.8) times.		One reported a completion rate of 58%.		One study reported high satisfaction.		One study reported a decrease in anxiety symptoms and stress symptoms.	
	LMICs (n=1)	One study reported an overall positive impression of the multimodal robotic system.		One study reported the need to adjust some features.		—		—	

^a^No evidence found.

^b^VR: virtual reality.

#### Web-Based Programs

Overall, 21 (32.3%; 5480/67,884, 8.1% out of total participants) out of the 65 studies included in this scoping review used web-based programs. In 17 studies that were conducted in high-income countries, a significant increase in registered users (n=2) and a high rating in usability (n=1) were reported. The completion rate ranged from 30.5% to 85% (n=5); the retention rate ranged from 30.5% to 85% (n=3); and the attrition rate was 13% (n=1). Quality checks of feasibility reported strong attendance (n=1), strong retentions (n=2), and high ratings of feasibility (n=2). Studies presented preliminary support for the feasibility of web-based programs yet with possible technical difficulty (n=1). In terms of acceptability, there was high satisfaction (n=5); the interventions were perceived to be acceptable, helpful, appropriate, and positive (n=3); and the acceptability scores were significantly better than average (n=1). Most studies, either when no control group is involved or when compared with the control group, reported improvements in anxiety symptoms and anxiety-related social impairment (n=8), while no effects on reducing anxiety were reported as well (n=2). The same patterns were found in outcomes measuring depression. Most studies reported improvements in depressive symptoms and depression severity (n=5), and there was no difference in depressive symptoms when the intervention group was compared with onsite groups (n=1). Improvements in stress-related symptoms (n=3), COVID-19–related worry (n=2), and insomnia (n=1) were identified.

In 4 studies conducted in LMICs, the mean time spent was 35.6 (SD 25.4) minutes (n=1) and the completion rate was 87.4% (n=1). High attendance and high retention rates (91.2%) were reported (n=1). With regard to acceptability, high ratings of acceptability and a high level of satisfaction (n=2) were reported. Studies, either when no control group is involved or when compared with the waitlist group, reported improvements in anxiety symptoms (n=4); some studies reported improvements in depressive symptoms (n=2), and 1 study reported reduction in stress and burnout.

#### Videoconferencing Platforms

Among the 65 studies, 16 (24.6%; participants: 1973/67,884, 2.9%) used teleconferencing platforms, including Zoom [30,39,49,55,59,60,62,72,73,81], Skype [25], Microsoft Teams [1,49], Google Meet [45], Tencent Meeting [19], and other platforms [41,42]. In 11 studies that were conducted in high-income countries, a completion rate between 92% and 100% (n=3), qualitative feedback of high perceived benefits (n=3), and technology-related challenges (n=3) were reported in terms of feasibility. High levels of satisfaction were reported (n=5). Studies reported improvements in depressive levels (n=9) and anxiety symptoms (n=5). No effects on decreasing anxiety symptoms were reported in one study. In 5 studies conducted in LMICs, the mean attendance ranged from 6.4 (SD 2.8) to 10.26 (SD 7.02; n=2). High levels of satisfaction (n=1) were reported. There were improvements in depressive symptoms and negative affect (n=3), yet no effects on decreasing depression levels were identified (n=2). No evidence was found in usability for LMICs or high-income countries.

#### Smartphone Apps

Of the 65 studies, 14 (21.5%; participants: 53,786/67,884; 79.2%) developed smartphone apps to deliver mental health services. In 11 studies conducted in high-income countries, studies reported high usability scores (n=2), and user engagement time ranged from 36.7 minutes over 12 weeks to 1424 minutes over 14 weeks (n=2). Feasibility was reported in terms of the average number of days retained (mean 42.4, SD 44.4; n=1), and the retention rate ranged from 28.3% to 85.7% (n=3). In terms of acceptability, the interventions were rated as “acceptable” (n=2), “satisfied” (n=2), “good” (n=1), and “helpful” (n=1). Studies reported a reduction in depressive symptoms compared with the preintervention and waitlist group (n=4). Similar improvements were reported in anxiety symptoms compared with preintervention, waitlist group, and a control app with limited access to psychoeducational contents (n=5). In 4 studies in LMICs, no evidence was found for usability and feasibility. Acceptability was reported as high satisfaction (n=1) and helpful and enjoyable (n=1). There were improvements in depressive symptoms, insomnia, psychological flexibility, and self-compassion, either when no control group is involved or when compared with the waitlist group (n=2).

#### SMS Text Messaging

Of the 65 studies, 5 (7.7%; participants: 5537/67,884, 8.1%) used the SMS text messaging technique. All studies were conducted in high-income countries. Studies reported high usability of ecological momentary intervention (n=1), and the completion rate ranged from 16% to 78%. An overall high satisfaction was reported (n=1). Studies suggested that an improved mood rating was observed at posttreatment assessments either with or without the control group (n=4). No evidence was found of studies conducted in LMICs for the technique of SMS text messaging.

#### Social Media

The use of social media as a technique to deliver mental health services was described in 3 (4.6%; participants: 116/67,884, 0.2%) out of 65 studies [44,54,79]. In one study conducted in high-income countries, high retention, high acceptability, and an effect on multiple outcomes (psychological distress, quality of life, functioning, loneliness, and physical activity) were reported (n=1). In the 2 studies conducted in LMICs, an average user time of 18.7 hours (n=1) was reported. Evidence suggested improvements in anxiety and depressive symptoms when compared with either usual care or the waitlist group (n=2). No evidence was found for other outcomes.

#### Hotline and Telephone Calls

Of the 65 studies, 3 (4.6%; participants: 886/67,884, 1.3%) reported using hotlines and telephone calls to deliver mental health services during the COVID-19 pandemic [36,47,65]. In 2 studies conducted in high-income countries, an average of 5.73 (SD 3.22) calls per day was reported (n=1). A dropout rate of 7.5% was reported (n=1), and there were improvements in loneliness, depression, anxiety, and general mental health, as reported in the same study (n=1). In one study that was conducted in LMICs, participants reported high satisfaction and mentioned that they would use the service again and would recommend it to others (n=1). No evidence was found in terms of usability, feasibility, or efficacy for the techniques of hotline and telephone calls by the studies conducted in LMICs.

#### Robotic Telemedicine and VR

Of the 65 studies, 3 (4.6%; participants: 106,67,884, 0.2%) used robotic telemedicine and VR. Two studies conducted in high-income countries reported an average use of 78 (SD 24.8) times (n=1) and a completion rate of 58% (n=1). High satisfaction was reported (n=1). Evidence suggested a decrease in anxiety symptoms and stress symptoms for all participants after the intervention (n=1). In 1 study conducted in LMICs, an overall positive impression of the multimodal robotic system was reported (n=1), yet the need to adjust some features was suggested (n=1). No evidence for acceptability or efficacy was found by studies conducted in LMICs.

### Meta-analyses

**Figure 2:**
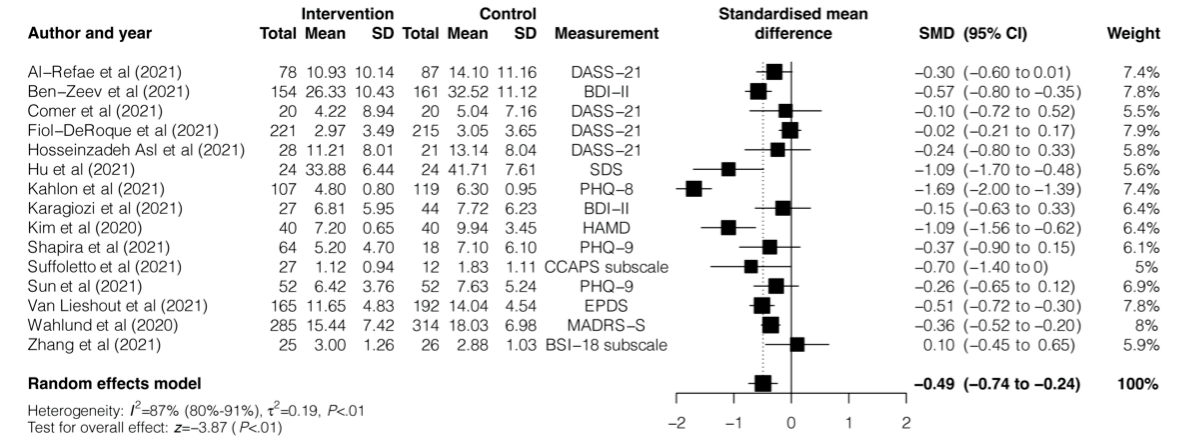
Effectiveness of digital mental health interventions in reducing depressive symptoms. BDI-II: Beck Depression Inventory-II; BSI: Brief Symptom Inventory; CCAPS: College Counseling Center Assessment of Psychological Symptoms; DASS-21: Depression Anxiety Stress Scales-21; EPDS: Edinburgh Postnatal Depression Scale; HAMD: Hamilton Rating Scale for Depression; MADRS-S: Montgomery Åsberg Depression Rating Scale-Self rated; PHQ: Patient Health Questionnaire; and SDS: Self-rating Depression Scale; SMD: standardized mean difference.

Of the 65 studies, 15 (23.1%) that reported depressive symptoms were included in the meta-analysis. Digital mental health interventions were associated with a small significant reduction in depressive symptoms (SMD −0.49, 95% CI −.74 to −.24; *P*<.001; Figure 2) with substantial heterogeneity (*I*^2^=87%, 95% CI 80%-91%; *Q*=107.57; *P*<.001).

**Figure 3:**
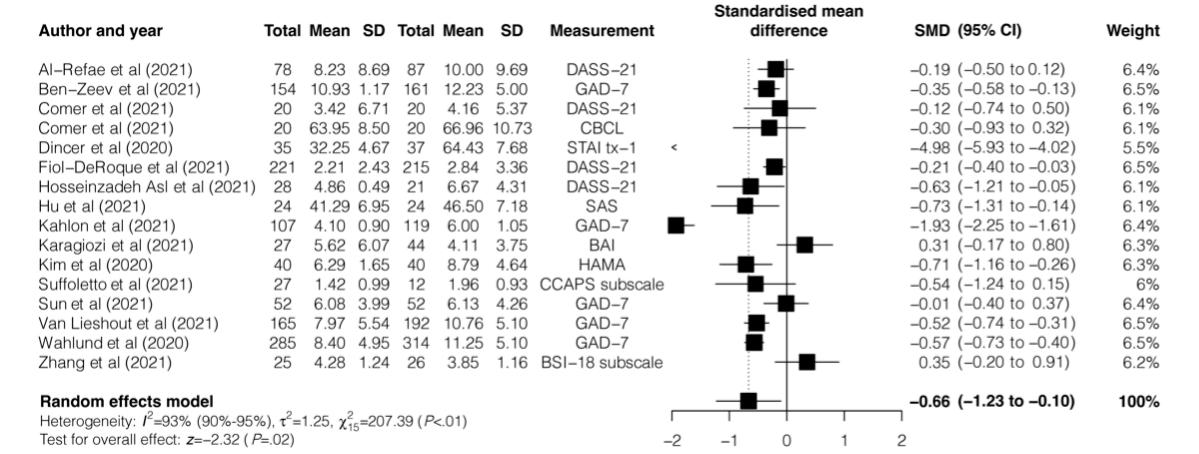
Effectiveness of digital mental health interventions in reducing anxiety symptoms. BDI-II: Beck Depression Inventory-II; BSI: Brief Symptom Inventory; CBCL: Child Behavior Checklist; CCAPS: College Counseling Center Assessment of Psychological Symptoms; DASS-21: Depression Anxiety Stress Scales-21; GAD-7: Generalized Anxiety Disorder-7; HAMA: Hamilton Anxiety Rating Scale; SAS: Self-rating Anxiety Scale; SMD: standardized mean difference; and STAI: State-Trait Anxiety Inventory.

The effectiveness of digital mental health interventions on anxiety symptoms was reported in 15 RCTs. The pooled SMD showed a moderate and significant effect of these interventions in reducing anxiety symptoms (SMD=−0.66, 95% CI −1.23 to −.10; *P*=.02; Figure 3) with substantial heterogeneity (*I*^2^=93%, 95% CI 90%-95%; *Q*=207.22; *P*<.001).

We examined the sources of heterogeneity by studying covariates across studies (Tables 3 and 4). Subgroup analyses revealed that reduced depressive symptoms were associated with the measurement of depression (using Depression Anxiety Stress Scales-21: SMD=−0.13, 95% CI −0.31 to 0.06 vs other measurements: SMD=−0.61, 95% CI −0.91 to −0.30; *Q*=7.04; *P*=.008). Reduced anxiety symptoms were not associated with sample size, location, measurement of anxiety, duration of follow-up, or the duration of intervention.

Meta-regression analyses are reported in Multimedia Appendix 4. There was no significant association between SMDs of depression and sample size, age, and proportion of females in the study population. Meta-regression analyses did not reveal differences in the SMDs of anxiety depending on sample size, age, and proportion of females in the study population.

For the main analyses of depression and anxiety, we observed evidence of funnel plot asymmetry, which could suggest no publication bias (Egger test: *P*=.57 and *P*=.50, respectively; Multimedia Appendix 5).

**Table 3:** Effect size of digital mental health services for reducing depressive symptoms during the COVID-19 pandemic according to study characteristics.

Group			Studies, n		SMD^a^ (95% CI)		*Q*		*P* value
**Sample size**							0.03		.86
	≥81	8		−0.51 (−0.86 to −0.16)					
	<81	7		−0.46 (−0.84 to −0.09)					
**Measurement of depression**							7.04		.008
	DASS-21^b^	4		−0.13 (−0.31 to 0.06)					
	Other measurements^c^	11		−0.61 (−0.91 to −0.30)					
**Location**							0.41		.52
	High-income countries	11		−0.53 (−0.83 to −0.24)					
	Low- and middle-income countries	4		−0.35 (−0.82 to 0.10)					
**Duration of follow-up**							1.23		.27
	Short-term	12		−0.55 (−0.84 to −0.26)					
	Long-term	3		−0.28 (−0.66 to −0.09)					
**Duration of intervention**							0.84		.36
	≥4 weeks	10		−0.56 (−0.90 to −0.23)					
	<4 weeks	5		−0.34 (−0.68 to −0.02)					

^a^SMD: standardized mean difference.

^b^DASS−21: Depression Anxiety Stress Scales-21.

^c^Other measurements include Beck Depression Inventory-II, Brief Symptom Inventory, College Counseling Center Assessment of Psychological Symptoms, Edinburgh Postnatal Depression Scale, Hamilton Rating Scale for Depression, Montgomery Åsberg Depression Rating Scale—Self rated, Patient Health Questionnaire, and Self-rating Depression Scale.

**Table 4:** Effect size of digital mental health services for reducing anxiety symptoms during the COVID-19 pandemic according to study characteristics.

Group		Studies, n	SMD^a^ (95% CI)	*Q*		*P* value	
**Sample size**					0.24		.62
	≥81	7	−0.54 (−1.01 to −0.07)				
	<81	8	−0.85 (−1.99 to 0.29)				
**Location**					0.49		.48
	High-income countries	10	−0.49 (−0.66 to −0.13)				
	LMICs^b^	5	−1.17 (−3.02 to 0.68)				
**Measurement of anxiety1**					0		.95
	GAD-7^c^	5	−0.68 (−1.31 to −0.04)				
	Other measurements^d^	10	−0.71 (−1.61 to 0.19)				
**Measurement of anxiety2**					2.06		.15
	DASS-21^e^	4	−0.23 (−0.38 to −0.08)				
	Other measurements	11	−0.85 (−1.68 to −0.02)				
**Duration of follow-up**					1.14		.29
	Short-term	12	−0.80 (−1.55 to −0.06)				
	Long-term	3	−0.29 (−0.86 to 0.29)				
**Duration of intervention**					0.58		.45
	≥4 weeks	9	−0.47 (−0.91 to −0.04)				
	<4 weeks	6	−1.07 (−2.56 to 0.41)				

^a^ SMD: standardized mean difference.

^b^LMICs: low- and middle-income countries.

^c^GAD-7: Generalized Anxiety Disorder-7.

^d^Other measurements including Beck Anxiety Inventory, Brief Symptom Inventory, Child Behavior Checklist, College Counseling Center Assessment of Psychological Symptoms, Hamilton Anxiety Rating Scale, Self-rating Anxiety Scale, and State-Trait Anxiety Inventory.

^e^DASS−21: Depression Anxiety Stress Scales-21.

### Risk of Bias Assessments

For the meta-analysis, we evaluated the risk of bias for the included RCTs, and the results are presented in Multimedia Appendix 6 [22,24,29,32,34,43,44,47-49,62,67,69,74,79,81]. Overall, the included studies showed low risk of bias. Of the 16 RCTs included, 6 (38%) studies were rated “low” risk; 7 (44%) studies were rated “some concerns”; and 3 (19%) studies were rated “high” risk. Owing to the absence of previously published analysis plans, bias in reporting was assessed as “some concerns” for all 16 studies.

## Discussion

### Principal Findings

To our knowledge, this is the most comprehensive review of the evidence examining the feasibility, acceptability, and efficacy of digital mental health services during the COVID-19 pandemic. The 65 studies identified in the scoping review reported using videoconferencing platforms, web-based programs, smartphone apps, SMS text messaging, social media, hotline, robotic telemedicine, and VR to deliver mental health services among 67,884 participants across 18 countries. Psychotherapy, psychoeducation, and psychological support were among the most common mental health services delivered during the infectious disease outbreak. Overall, digital mental health services delivered during the initial phase of COVID-19 were usable, feasible, and acceptable and improved psychological well-being in participants across the included studies in high-income countries and LMICs. Sixteen RCTs that reported comparable outcomes, including for depression or anxiety, were systematically reviewed and meta-analyzed. The results indicated that digital mental health interventions were significantly associated with a small reduction in depressive symptoms and a moderate reduction in anxiety symptoms. The significance and effect sizes of the interventions differed among the measurements of the depression.

Our study suggested that the feasibility, acceptability, and effectiveness of digital mental health services during the pandemic were similar to those before the pandemic [82,83], indicating the potential for digital intervention to respond to the mental health needs of the people during the pandemic. These needs include addressing mental health problems among people experiencing COVID-19–related symptoms of anxiety, depression, and stress; patients with suspected or confirmed COVID-19; health workers; and patients with preexisting mental disorders. The advantages of digital mental health services in promoting psychological well-being depend on the ability to overcome the physical distance between people with mental health needs and providers and increasing the accessibility of mental health services. Although the use of digital mental health services has long been advocated, it has not yet been widely adopted because of a lack of support from both service users and health care professionals. In most nations, the situation has changed after the COVID-19 pandemic [84]. In China, for example, increased use of web-based mental health assessments, psychoeducation, and psychological counseling were observed during the COVID-19 pandemic [85].

Different strategies should be used for applying suitable techniques to digital mental health services. In our review, we found that smartphone app outreach was used by most service users (79.2%). Individuals with mild to moderate mental health symptoms, which may be addressed by psychosocial support and psychological interventions based on a stepped-care approach, are the target populations for the expanded use of smartphone apps to improve the delivery of mental health services. The technology with broad reach could also be used for psychoeducation [46], which is necessary for the support and prevention of mental disorders. Previous research has found that using apps or other web-based treatments for the older adult population can be challenging. Our study found that older adults have positive attitudes toward and may benefit from the electronic device–based mental health services, including videoconferencing platforms [45,62], social media [54], and web-based programs [77]. We suggest that smartphone apps and other web-based interventions should be considered for the older age population if technical assistance could be provided for people with difficulties with these devices. Telephone calls that are easy to use should also be considered for older adults [47].

The use of digital mental health services was reported across high-income countries and middle-income countries, but there were no studies from low-income countries. Digital mental health services have great potential in LMICs because of the limited availability of mental health services to cover the population needs [86] along with diminished health system capacity during the pandemic. Promising findings were reported for digital mental health services (videoconferencing platforms, web-based programs, and smartphone apps) in most high-income countries and some middle-income countries. However, these techniques rely on the internet and may be difficult to replicate in low-income areas. In addition, limited studies have examined the use of SMS text messaging and telephone calls in middle-income countries. Techniques that do not rely on the internet are a priority in low-resource areas. For instance, owing to their great accessibility and low cost of use, SMS text messaging and hotline might be considered for use in resource-limited or isolated locations. The benefits of delivering digital mental health services in remote and low-resource areas, in terms of potential impact and resource savings, must be considered alongside the feasibility of achieving high coverage of mental health services.

The finding that digital mental health services are associated with the amelioration of anxiety and depressive symptoms is consistent with previous evidence examining their effects [9,87]. Subgroup and meta-regression analyses allowed us to identify the effects of the study and population characteristics. For instance, there was no evidence of differences in SMD based on sample size. We found that depression measures contributed to heterogeneity. The posttreatment effects of digital mental health interventions differed from Depression Anxiety Stress Scales-21 (DASS-21) and other measurements. Although the current evidence supports the short-term effectiveness of most digital mental health services, a few studies have evaluated long-term outcomes after the interventions [43,69,79]. The long-term benefits of receiving digital mental health services should be assessed through practical evaluations. Along with the viability of reaching high coverage, it is important to weigh the potential impacts and resource savings of providing digital mental health care in rural and underresourced regions.

Our review sheds light on future research. First, further efforts should be focused on how digital mental health services can be integrated with existing traditional treatment modalities to form a complementary and integrated service model. In particular, the integration of medication and digital services remains to be further validated for safety and feasibility. Second, most studies included in our review were digital psychotherapy interventions, suggesting that digital technologies may have facilitated the accessibility of psychotherapy during the pandemic. We found evidence supporting the efficacious delivery of psychotherapies, either guided [62,77] or self-help therapy [31,34,64,74]. In a study by Al-Alawi et al [18], therapist-guided web-based therapy was associated with greater effectiveness than self-help therapy for people with anxiety and depressive symptoms during the COVID-19 pandemic. Future studies should focus on determining whether people require a guided web-based psychotherapy approach and promote individualized treatment to ensure the optimal allocation of treatment resources. Third, despite the diversity of mental disorders, only a few are covered in this review. Depression and anxiety were the most common outcomes measured. Thus, the role of digital services for many severe mental illnesses, including schizophrenia and bipolar disorder, needs to be further examined.

Our study has implications for policy development in health care. During a pandemic, digital mental health technology can be used as a backup to address psychological demands and broaden service coverage. We urge policy makers to create future industry regulatory laws so that the digital health sector can grow in a way that provides security and effectiveness testing. The development of digital health infrastructure, including information transmission facilities and technical testing methods for data security issues, is also a crucial prerequisite for the future development of digital mental health. If digital mental health services are to continue, there is a need to specify the minimum level of privacy and security that is acceptable. For example, in the United States, digital mental health services have rapidly made changes in response to policies and regulations concerning confidentiality and privacy to promote telehealth delivery of care [88].

### Limitations

Our study has several limitations that should be noted. First, owing to the nature of our research question, the scoping review demonstrated substantial heterogeneity. In the pool of included studies, there was a high heterogeneity in terms of study design, type of techniques, type of mental health services, intervention components, target conditions, populations, and outcomes. Most studies did not have comparisons, and their effectiveness remains to be examined in future studies. For meta-analyses, we found only a few factors that contribute to heterogeneity between studies. The subgroup analyses were based on outcomes and limited by the variations in the type of techniques and type of mental health services. As a result, the findings should be interpreted with caution and read in terms of implications for future research.

Second, most participants in our scoping review were from high-income nations, primarily the United States and European countries. There is an urgent need for more independent research that examines the viability, acceptability, and efficacy of digital mental health as a response to pandemics in LMICs, particularly in low-income countries.

Third, although the current evidence supports the short-term effectiveness of most digital mental health services, only 2 studies have evaluated the long-term outcomes. A practical evaluation of the long-term benefits of digital mental health services should be undertaken, particularly in urban contexts.

Fourth, this study did not include preprint servers in the search, which may lead to the overestimation of our findings. However, a methodological research study of systematic reviews and meta-analyses revealed that including unpublished data (such as preprints) may add new sources of bias, although it does not change the results of reviews [89]. In addition, there is a possibility that the findings in preprints will be changed further, which leads to the results of the review being amended as well.

Finally, the target of this study was to evaluate the usability, feasibility, acceptability, and initial efficacy of digital mental health services in the initial phase of the pandemic. Therefore, we limited the data search to the first 2 years of the pandemic. We recommend that future studies should assess whether these services will remain the same or be further refined during other phases of the pandemic.

### Conclusions

In summary, we have reported a range of digital mental health services delivered during the initial phase of the COVID-19 pandemic. We found that digital mental health services were usable, feasible, acceptable, and effective in response to mental health needs in the initial phase of pandemic across high-income countries and middle-income countries. Our findings also highlight the amelioration of depressive and anxiety symptoms based on the pooled results of the RCTs. To date, the strategies to lessen the COVID-19 pandemic’s negative impacts on mental health remain a subject of much debate. We suggest that policy makers develop, implement, and assess different strategies for addressing the long-term mental health needs of the pandemic and its aftermath. Digital mental health services as novel strategies for promoting psychological well-being can purposively address physical barriers and provide a promising element for future integrated mental health service models in the post–COVID-19 era.

## Appendix

Multimedia Appendix 1 PRISMA (Preferred Reporting Items for Systematic Reviews and Meta-Analyses) checklists.

Multimedia Appendix 2 Search databases and strategy.

Multimedia Appendix 3 Summary of the findings of individual studies included in the qualitative systematic review.

Multimedia Appendix 4 Results of the meta-regression for depression and anxiety.

Multimedia Appendix 5 Publication bias of meta-analyses.

Multimedia Appendix 6 Risk of Bias across randomized studies for meta-analyses.

## Abbreviations

CBT: cognitive behavioral therapy
DASS-21: Depression Anxiety Stress Scales-21
LMICs: low- and middle-income countries
PRISMA: Preferred Reporting Items for Systematic Reviews and Meta-Analyses
PRISMA-ScR: PRISMA Extension for Scoping Reviews
RCT: randomized controlled trial
SMD: standardized mean difference
VR: virtual reality
