# Supplementary material for: The Usability, Feasibility, Acceptability, and Efficacy of Digital Mental Health Services in the COVID-19 Pandemic: Scoping Review, Systematic Review, and Meta-analysis
Source: JMIR Public Health Surveill. 2023 Feb 13;9:e43730. doi: 10.2196/43730 (PMC9930923; doi:10.2196/43730)
Supplement: Multimedia Appendix 2 [file publichealth_v9i1e43730_app2.docx]

**Multimedia Appendix 2. Search databases and strategy**

**Search databases and results**

| **Databases** | **Records** |
| --- | --- |
| EMBASE | 4260 |
| PUBMED | 2630 |
| PSYCINFO | 579 |
| COCHRANE | 37 |
| **TOTAL** | **7506** |

**Search terms**

| #1 | “serious mental illness” OR “severe mental illness” OR “mental illness” OR “mental health” OR “mental disorder” OR “psychotic disorders” OR “psychosis” OR “schizophrenia” OR “schizoaffective disorder” OR “bipolar disorder” OR “affective disorder” OR “mood disorders” OR “hypomania” OR “mania” OR “manic depression” OR “manic-depression” OR “cyclothymic disorder” OR “major depressive disorder” OR “depression” OR “anxiety” OR “post-traumatic stress disorder” OR “ptsd” OR “stress disorder” OR “substance use” OR “substance use disorder” OR “substance related disorder” OR “alcohol” OR “alcohol use disorder” OR “alcoholism” OR “drug abuse” OR “amphetamine” OR “cocaine” OR “crack cocaine” OR “marijuana” OR “cannabis” OR “opioid” OR “heroin” OR “suicide” OR "mental disorders"[MeSH Terms] OR "Schizophrenia and Disorders with Psychotic Features"[Mesh] OR "Psychotic Disorders"[Mesh] OR "Anxiety Disorders"[Mesh] OR "Stress Disorders, Traumatic"[Mesh] OR "Mood Disorders"[Mesh] OR"Bipolar Disorder"[Mesh] OR "Depressive Disorder"[Mesh] OR "Substance-Related Disorders"[Mesh] OR "Alcohol-Related Disorders"[Mesh] OR "Amphetamine-Related Disorders"[Mesh] OR "Cocaine-Related Disorders"[Mesh] OR "Marijuana Abuse"[Mesh] OR "Opioid-Related Disorders"[Mesh] OR “suicide”[Mesh] |
| --- | --- |
| #2 | “telemedicine” OR “telepsychiatry” OR “telehealth” OR “telecare” OR “telemental health” OR “telemetry” OR “electronic health” OR “eHealth” OR “uHealth” OR “mHealth” OR “Connected Health” OR “online Intervention” OR “Internet-based intervention” OR “Internet health” OR “web-based intervention” OR “social media” OR “Facebook” OR“Wechat” OR “Weibo” OR “microblog” OR “mobile health” OR “mobile technology” OR “mobile phone” OR “mobile devices” OR “mobile health technologies”OR “Cellular Phone” OR “Cellphone”OR “smartphone” OR “smartphone application” OR “smartphone app” OR “smartphone technology” OR “text message” OR “SMS” OR “short message service” OR “text messaging” OR “artificial intelligence” OR “digital health” OR “digital medicine system” OR “wearable device” OR “virtual reality” OR “virtual reality intervention” OR “computer-assisted therapy” OR “Therapy, Computer-Assisted” OR “Remote Sensing Technology” OR “Remote consultation” OR "Telemetry"[Mesh] OR "Telemedicine"[Mesh] OR "Internet-based Intervention"[Mesh] OR "Social Media"[Mesh] OR “mobile application”[Mesh] OR “smartphone”[Mesh] OR "Cell Phone"[Mesh] OR "Text Messaging"[Mesh] OR "Artificial Intelligence"[Mesh] OR "Therapy, Computer-Assisted"[Mesh] OR "Remote Sensing Technology"[Mesh] |
| #3 | "coronavirus" OR "corona virus" OR "betacoronavirus" OR "beta coronavirus" OR "2019-nCoV" OR "nCovor " OR "SARS-CoV-2" OR "SARS CoV 2" OR "severe acute respiratory syndrome coronavirus" OR " coronavirus" OR " nCov" OR "cov2" OR "sars 2" OR "COVID" OR "COVID-19"OR"Coronavirus infections"[Mesh] OR "COVID-19"[Mesh] OR "SARS-CoV-2"[Mesh] |
