# Supplementary material for: The Usability, Feasibility, Acceptability, and Efficacy of Digital Mental Health Services in the COVID-19 Pandemic: Scoping Review, Systematic Review, and Meta-analysis
Source: JMIR Public Health Surveill. 2023 Feb 13;9:e43730. doi: 10.2196/43730 (PMC9930923; doi:10.2196/43730)
Supplement: Multimedia Appendix 3 [file publichealth_v9i1e43730_app3.docx]

**Multimedia Appendix 3. Summary of the findings of individual studies included in the qualitative systematic review.**

**Table S1. Summary of findings of individual study included in the qualitative systematic review by type of techniques**

| **Author (Year)** | **Location** | **Study period** | **Target population** | **Study aim** | **types of intervention and components** | **Summary of interventions** | **Findings** | **Measurements** |
| --- | --- | --- | --- | --- | --- | --- | --- | --- |
| ***Videoconferencing platforms*** | | | | | | | | |
| Bantjes (2021)[23] | South Africa | - | university students | to investigate the uptake, retention, treatment response, and level of satisfaction with a remote group CBT intervention for university students during the COVID-19 pandemic. | psychotherapy; Group CBT | The intervention was delivered via Microsoft Teams in 10 weekly workshops of 60-75 minutes. The content was organized into 5 themes, with each theme spanning 2 workshops. Five themes were included: You feel the way you think; Planning to succeed; Hacks to boost your mood; Building mastery; Avoiding meltdowns | **feasibility:** The mean number of sessions attended was 6.4 (SD 2.8) out of 10.  **acceptability**: High overall levels of satisfaction with treatment were reported. **effectiveness**: mean symptom scores decreased significantly for anxiety (P<.001), depression (P<.001), and composite anxiety and depression (P<.001), with large effect sizes (d=1-1.5). | **feasibility** : mean number of session attendance.  **acceptability**: Client Satisfaction with Treatment Questionnaire;  **effectiveness**: GAD-7 and PHQ-9 |
| Brouzos (2021)[25] | Greece | April 22 to May 8, 2020 | adults | to investigate the effectiveness of a group online positive psychology intervention designed to mitigate the psychological impact of the COVID-19 pandemic | psychotherapy; positive psychology group intervention that incorporated some elements of CBT | The intervention was delivered online using various teleconferencing free software (e.g., Skype) in small groups of 5–7 members. Each session lasted approximately 50 minutes and the intervention’s duration was two weeks (three sessions per week). | **effectiveness**: The intervention was found to be effective in alleviating the impact of the pandemic and in strengthening participants’ resilience. More specifically, the results showed significant decreases for the intervention group in all measures of psychosocial distress (anxiety, depression, loneliness and fear) and significant increases in empathy, resilience, and experience of positive emotions. | **effectiveness**: Interpersonal Reactivity Index, Connor-Davidson Resilience Scale, Positive and Negative Affect Schedule, De Jong Gierveld Loneliness Scale, GAD-7, PHQ-9 |
| Craig (2021)[30] | Canada | May 15 to 15 September 2020 | LGBTQA+ Youth and Young Adults | This study describes the preliminary efficacy of AFFIRM Online | psychotherapy; CBT | A total of 8 AFFIRM Online groups (which consisted of eight weekly sessions) were delivered with 6–14 distinct participants in each group. The curriculum includes an orientation (group norms, confidentiality, curriculum); sessions 1–2 (overview of CBT and the impact of minority stressors); sessions 3–4 (CBT, thought stopping); sessions 5–6 (coping skills, goal setting and cultivating hope); and sessions 7–8 (social support, self-compassion, self-care plans) | **acceptability**: participants reported high acceptability; **effectiveness**: Compared to a waitlist control, AFFIRM Online participants experienced significantly reduced depression (P=.005, d=0.60) and improved appraisal of stress as a challenge (P=.005, d=0.60) and having the resources to meet those challenges (P=0.059, d=0.39) as well active coping (P=.012, d=0.54), emotional support (P=0.017, d=0.51), instrumental support (P<.001, d=0.77), positive framing (P=.046, d=0.42), and planning (P=.024, d=0.49). | **acceptability**: AFFIRM Acceptability Survey;  **effectiveness**: BDI-II; the Brief COPE Inventory; Proactive Coping Inventory for Adolescents-A; Reflective Coping Subscale; Stress Appraisal Measure for Adolescents; Hope Scale |
| Guan (2021)[19] | China | end of December 2020 | college students | to investigate online self-compassion exercises' effectiveness in alleviating people's negative affect during the COVID-19 pandemic lockdown | psychotherapy; three components of self-compassion: mindfulness, common humanity, and self-kindness | participants completed a writing task that contained a series of writing prompts that aimed to induce the three components of self-compassion: mindfulness, common humanity, and self-kindness. | **effectiveness**: there were significant increases in participants' self-compassion and decreases in negative affect when compared to participants in the control condition | **effectiveness**: state self-compassion scale-long form, STAI, negative emotion subscale of the positive and negative affect schedule |
| Guo (2020)[40] | China | early March 2020 | caregivers of patients with eating disorders | to assess depression and anxiety levels among caregivers of patients with ED in China, and assess whether an online education program, as a low-cost, safe, and convenient form of intervention, is effective in decreasing the anxiety and depression of these caregivers in the context of the serious pandemic. | psychotherapy; health education | The online education program lasted for 4 weeks and a total of four ED related lectures for caregivers were given through the Zoom software. The lectures were delivered by different therapists each time at a frequency of once a week. In each lecture, a specific topic on skills dealing with children or adolescents' ED was discussed and explained by the therapist for 1 hr, followed by a 30-minute question-and-answer session. After each lecture, participants were invited to write down their thoughts on the lecture and their experience in fighting against ED. | **effectiveness**: the online education program showed no significant effect on decreasing depression and anxiety levels of caregivers of patients with ED overall | **effectiveness**: PHQ-9; GAD-7; |
| Held (2021)[41] | US | not specified | veterans of PTSD | to provide an overview of the development of a 2-week virtual intensive PTSD treatment program | psychotherapy; CPT, mindfulness, art therapy, DBT | 18 sessions of individual CPT over the course of the 2-week vITP. Veterans received two individual CPT sessions per day, starting on the first day of treatment. The DBT-based skills group curriculum was covered over five 50-minute sessions and included the following five skills: crisis skills, emotion education, emotion regulation skills, healthy relationship boundaries, and keeping and improving relationships/maintaining self-respect. In the afternoons, veterans had the option to participate in mindfulness sessions, guided instruction in trauma-sensitive yoga, as well as art therapy. | **feasibility**: veteran 1 and 2 utilized all 18 individual CPT sessions; both therapists endorsed being pleasantly surprised by the ease of use and lack of technological disruptions related to the virtual platform; both therapists had positive experiences utilizing telehealth for this vITP and were encouraged by how effective treatment could be delivered through this format; **effectiveness**: veteran 1 showed a slow, steady decline in PTSD symptoms over the course of treatment. Veteran 1 reported a 19-point reduction in PTSD symptoms and a 1-point reduction in depressive symptoms. Veteran 2 reported a 48-point reduction in PTSD symptoms and a 16-point reduction in depression symptoms | **feasibility**: completion, therapists opinion; **effectiveness**: PCL-5, PHQ-9 |
| Hom (2020)[42] | US | March 25 to April 15, 2020 | an acute psychiatric population | to present preliminary evidence of our virtual PHP’s acceptability and feasibility | psychotherapy; CBT, DBT, ACT | 15 CBT/DBT/ACT groups/week (3 groups/day); 3 CTM meetings/week for case management, aftercare planning, and individualized treatment planning; 2-3 psychiatrist meetings/week for med. Management; 3 individual therapy sessions/week for skills work; 1-2 vocational counseling sessions, as needed; 1 family meeting encouraged | **feasibility**: daily census has steadily increased over time, from an average of seven patients during the fifirst quarter of our active program to an average of 10 to 11 patients during the most recent quarter; **acceptability**: Patients have reported group sessions and individual meetings to be exceedingly helpful. Patients who have discharged thus far have also expressed confidence in their aftercare plans. The two patients who expressed having very positive experiences with the virtual PHP. On a scale of 1 to 10, with 10 being the highest rating, both rated the care they received as a 9. | **feasibility**: attendance and daily census; **acceptability**: a rating scale of 1 to 10 on satisfaction, opinions |
| Ibrahim (2021)[45] | Malaysia | May and June 2020 | aged 60 years and over | conducted a feasibility study to evaluate recruitment, data collection and group exercise intervention delivered through virtual technology among individuals aged 60 years and over in Malaysia | virtual group exercise; Physical activities | A four-week course of virtual group exercise; participants were invited to take part in a 30-min daily virtual exercise class; started with deep breathing and correction of posture; the sessions ended with deep breathing and a reminder to fifill-in their exercise diary | **feasibility**: overall mean attendance was 10.26 (SD=7.02) sessions. Most participants attended 14 sessions (n = 8) and one attended all the 20 sessions.  **effectiveness**: no significantly difference between groups and across time in anxiety and depression scores | **feasibility**: attendance;  **effectiveness**: HADS |
| Kim (2020)[49] | Malaysia | not specified | adults | to assess the effect of Tele-acupressure self-practice on the improvement of mental health as well as depression, anxiety and well-being during COVID-19 | physical therapy; acupressure | The Tele-acupressure self-practice group received live stream acupressure self-practice with the following stages: (1) explain the acupressure points location, self-practical skills for acupressure, and the start of each acupressure point was self-pressed for 2 minutes following the lecturer; (2) warm-up acupressure point was self-practiced for 5 minutes following the lecturer; (3) acupressure self-practice for no longer than 40 minutes following the lecturer. For each participant, acupressure self-practice was conducted once everyday for four weeks. All Tele-acupressure self-practice education was conducted via live stream by a trained lecturer. | **effectiveness**: the Hamilton Depression Rating Scale (6.29±1.65), Hamilton Anxiety Rating Scale (7.20±0.65), and anxiety score in well-being ONS-4 (3.34±0.38) were significantly lower; the happiness score in well-being ONS-4 (8.82±0.86) was significantly higher in the Tele-acupressure self-practice group compared with the non-acupressure group in the after four week’s assessments | **effectiveness**: HAMD, HAMA |
| Nauphal (2021)[55] | US | early April to mid-June 2020 | adults with social anxiety disorder | to examine the acceptability, feasibility, and preliminary effectiveness of an adapted telehealth-delivered group CBT intervention for adults with social anxiety disorder | psychotherapy; CBT, mindfulness, social self-reappraisal therapy | eight two-hour sessions, including Orientation to Telehealth and Psychoeducation (Session 1), Cognitive Flexibility and Mindfulness (Session 2), Exposures (Sessions 3–7), Treatment Review and Relapse Prevention (Session 8) | **feasibility**: all group members were highly engaged in the treatment;  **acceptability**: high overall satisfaction to participants;  **effectiveness**: reduction in symptoms of social anxiety, general anxiety, depression, and stress | **feasibility**: completion; **acceptability**: CSQ; Telemedicine Satisfaction and Acceptance Scale; **effectiveness**: Social Phobia Inventory; Overall Anxiety Severity and Impairment Scale; Overall Depression Severity and Impairment Scale; DASS-21 |
| Puspitasari (2021)[59] | US | March to August 2020 | adults (aged 16-65 years) who are at risk for psychiatric hospitalization | to evaluate the feasibility and initial effectiveness of the Adult Transitions Program, a group-based teletherapy intensive outpatient program for adults with transdiagnostic conditions who are at risk for psychiatric hospitalization | comprehensive intervention (intensive psychiatric outpatient program); multiple components: CBT, behavioral activation, dialectical behavior therapy | The ATP video teleconferencing format was delivered 5 days per week, 3 hours per day, and it consisted of primarily group-based interventions with rolling admission. The program consisted of three tracks, with 8 patients in each track at any given time. In addition to receiving three group teletherapy sessions per day, patients also received individual sessions throughout their enrollment in the program. | **feasibility**: 70 of the 76 patients (92%) completed the program with a mean attendance of 14.43 days (SD 1.22); 71 patients (95%) scheduled at least one behavioral health aftercare service prior to ATP discharge;  **effectiveness**: significant improvements in depression (Cohen d=0.77; P<.001), anxiety (d=0.74; P<.001), overall suicide risk (d=0.41; P=.02), wish to live (d=0.39; P<.001), wish to die (d=0.52; P=.01), and overall mental health (d=0.39; P<.001) from admission to discharge. | **feasibility**: number of patients who completed the program, average days of attendance, and securing of behavioral health aftercare services prior to ATP discharge **effectiveness**: PHQ-9, GAD-7, Suicide Status Form |
| Rojas (2021)[60] | US | 2020 | adult veteran who had acute suicide risk before or during hospitalization | to examine the delivery of brief CBT for suicide prevention (BCBT-SP) via Clinical Video Telehealth (CVT) to the home of a veteran discharged from the psychiatric inpatient unit after a recent suicide attempt | psychotherapy; brief CBT | Brief CBT for suicide prevention uses a phased treatment approach (i.e., Phases 1–3) to directly target suicide risk. The treatment lasts for approximately 12 sessions: phase 1 focused on deactivation of the suicidal mode by targeting behavioral risk factors via crisis management and building emotion regulation skills; phase 2 aimed to undermine the suicidal belief system by targeting cognitive risk factors and reinforce engagement in value-driven activities; phase 3 was dedicated to the relapse prevention task | **feasibility**: the veteran successfully completed the third module of BCBT-SP in 9 sessions; one technical failure; **acceptability**: high perceived therapeutic alliance and high acceptability; **effectiveness**: the veteran did not engage in any suicidal behavior during treatment, and suicidal ideation, depression, and anxiety decreased as treatment progressed reinforcing | **feasibility**: retention, technical failure;  **acceptability**: helping alliance questionnaire–II, telehealth usability questionnaire, qualitative feedback;  **effectiveness**: Columbia–Suicide Severity Rating Scale; DASS-21 |
| Shapira (2021)[62] | Israel | April to June 2020 | community-dwelling older (65+) adults | to explore the efficacy of a short-term digital group intervention aimed at providing seniors with the tools and skills necessary for improving their coping ability during these stressful times | psychotherapy; CBT (mainly), mindfulness | The intervention included seven twice-weekly online guided group sessions via Zoom; each session lasted between 60 and 90 min, and consisted of a guided group discussion (20–30 min), and learning and practicing cognitive-behavioral techniques and skills; cognitive schemas, cognitive restructuring, and constructing positive self-talk (lasted 40–60 min); mindfulness techniques were taught as well | **effectiveness**: a significant improvement in the intervention group in terms of both loneliness and depressive symptoms, compared with the control group; Results of mixed effect models indicated a medium ameliorative effect on loneliness (d=0.58), while that for depressive symptoms was only marginally significant and smaller in size (d=0.43) | **effectiveness**: UCLA loneliness, PHQ-9 |
| van Agteren (2021)[72] | Australia | recruit from March to July 2020 | adults (18 years or older) | to investigate the differential impact of an internet-based group mental health intervention on outcomes of positive mental health and indicators of psychological distress | psychotherapy; mindfulness, self-compassion, stress, coping, and resilience | The “Be Well Plan” is a 5-week, group-based psychological program that helps non-clinical participants create a personalized mental health and well-being plan by experimenting with a variety of resources and evidence-based activities to improve mental health and well-being; provided 5 2-hour weekly sessions hosted online via Zoom | **effectiveness**: significantly improved all mental health outcomes measured, including mental health well-being and psychological distress (P<.001) | **effectiveness**: Mental Health Continuum Short-Form; Satisfaction with Life Scale, DASS-21, Brief Resilience Scale |
| Van Lieshout (2021)[81] | Canada | April 20 to October 4, 2020 | women had an infant and had an EPDS score of at least 10 | to determine whether self-referred online 1-day CBT-based workshops for Postpartum depression plus treatment as usual could improve PPD compared with TAU alone | psychotherapy; CBT | a 1-day interactive workshop consisting of didactic teaching, group exercises/ discussion, and role-playings; workshops took place every 3 weeks | **effectiveness**: The workshop led to significant mean (SD) reductions in EPDS scores (P<.001) and GAD-7 scores (P<.001) | **effectiveness**: EPDS; GAD-7 |
| Wagner (2020)[73] | US | not specified | children under three years of age and clinicians | to examine the acceptability and the feasibility of the TELE-ASD-PEDS as a tool for tele-assessment | psychiatric assessments and interviews; diagnosing ASD | The TELE-ASD-PEDS is a novel tool developed for the remote observation of autism-related behaviors, to augment diagnostic decision-making by an expert clinician; includes eight discrete, caregiver-led activities or social bids, including opportunities for interactive play, physical play routines, and requesting activities | **feasibility**: all providers experienced technology-related challenges and perceived benefits; Some providers reported challenges related to the home environment, obtaining and documenting informed consent for tele-assessment procedures, and the use of the TELE-ASD-PEDS **acceptability**: clinicians felt satisfied and comfortable; most reported the tool was appropriate and have benefits during restriction and in terms of limiting travel and transportation barriers | **feasibility**: qualitative feedback; **acceptability**: provider survey |
| ***Web-based programs*** | | | | | | | | |
| Al-Alawi (2021)[18] | Oman | recruited between April 14 to May 30, 2020 | patients with COVID-19–induced symptoms of anxiety and depression | to investigate and comparatively assess the efficacy of a 6-week-long therapist-guided online therapy course with that of internet-based (email-delivered), self-help therapy focusing on COVID-19–related symptoms of anxiety and depression among individuals in Oman during the COVID-19 pandemic | psychotherapy; CBT and ACT | 1 online session per week for 6 weeks from certified psychotherapists; the psychotherapists utilized CBT and acceptance and commitment therapy interventions | **effectiveness**: a significant reduction in the GAD-7 scores (P=.01) between the two groups after adjusting for baseline scores. GAD-7 scores of participants in the intervention group were considerably more reduced than those of participants in the control group (P=.01). A greater reduction in mean PHQ-9 scores was observed among participants in the intervention group (P=.006) than those in the control group (P=.006); the levels of anxiety and depression reduced in both study groups, but was higher in the intervention group (P=.049) than in the control group (P=.02) | **effectiveness**: GAD-7; PHQ-9 |
| Casas (2021)[26] |  |  |  | to assess the feasibility and acceptability of the Written Exposure Therapy protocol delivered via telehealth | psychotherapy; Written Exposure Therapy | participants completed five sessions of the intervention by following the Written Exposure Therapy protocol for five consecutive weeks | **feasibility**: five participants (33.3%) left treatment prior to the completion of the treatment protocol and ten participants (66.7%) completed treatment. **acceptability**: participants endorsed that they actually felt that the treatment is credible and expected to achieve an improvement in their symptoms. **effectiveness**: a mean reduction of 16.20 points on the PCL-5, a statistically significant difference in symptom severity from pre- to post- treatment (P<.01).There was a statistically significant difference between session one and session five in terms of self-reported emotional valence on the SAM (P<.001), with participants experiencing a one and a half category positive change in emotional valence | **feasibility**: completion; **acceptability**: Treatment Expectancy Questionnaire, CSQ-8, Working Alliance Inventory Short Form, **effectiveness**: PCL-5, Self-Assessment Manikin |
| Comer (2021)[29] | US | not specified | children with social anxiety disorder and their parents | to examine the preliminary efficacy of a family-based behavioral parenting intervention | psychotherapy; parent-child interaction therapy | a 12-session manual-based modification of parent-child interaction therapy for treating anxiety in early childhood. Families were given 16 weeks to complete treatment. | **feasibility**: iCALM Telehealth Program was well tolerated by families, with 85% of families completing their course of treatment. **acceptability**: families reporting very high satisfaction with services: 82.6% reported “very satisfied” with the services received, 87.0% rated the quality of the services received as “excellent,” and 91.3% of treated families reported that they would “definitely” recommend the program to a friend if they were in need of similar help. **effectiveness**: significantly greater reductions than waitlist in child anxiety symptoms, fear, discomfort, and anxiety-related social impairment, and also led to greater improvements in child soothability; particularly effective in reducing life impairments and parental distress among families presenting with higher, relative to lower, levels of baseline parental accommodation | **feasibility**: treatment retention； **acceptability**: CSQ-8; **effectiveness**: Child Behavior Checklist; parent-report Children’s Behavior Questionnaire Short Form; Child Anxiety Impairment Scale; DASS-21 |
| Detweiler Guarino (2021)[31] | US | April to October 2020 | general population | to assess the acceptability and usability of the PATH content and determine whether such a program would be useful as a stand-alone open-access resource | psychotherapy; CBT | the program contains three primary modules: conflict management, stress management, and depression treatment. Users were able to browse the program’s website freely and to choose to go through the cognitive-behavioral modules they selected at their own pace. | **usability**: 2484 users registered; 562 individuals started the program; **feasibility**: The attrition rate was high with 13% (21/156) completing the conflict module, 17% (50/289) completing session one of the mood module, and 14% (16/117) completing session one of the stress module;  **acceptability**: acceptability scores for the mood and stress modules were significantly better than average; **effectiveness**: symptoms of stress showed a significant improvement over time (P=.03), and there was a significant decrease in depressive symptoms over all time points (P=.01); depression severity decreased on average by 20% (SD 35.2%; P=.60) between sessions one and two | **usability**: numbers of individuals who registered and started the program; **feasibility**: attrition rate; **acceptability**: Acceptability of Self-Guided Treatment; **effectiveness**: PHQ-9, PSS |
| Dincer (2020)[32] | Turkey | May 2020 | nurses caring for COVID-19 patients in a university hospital | to investigate the efficacy of emotional freedom techniques in the prevention of stress, anxiety, and burnout in nurses | psychotherapy; Emotional Freedom Techniques | Emotional Freedom Techniques were applied to each group of nurses in a single session of approximately 20 min. The EFT session began by presenting the participants with a picture of the acupressure points and showing them how to gently tap on them using their index and middle fingers. | **effectiveness**: reductions in stress (P<.001), anxiety (P<.001), and burnout (P<.001) reached high levels of statistical significance for the intervention group. The control group showed no statistically significant changes on these measures (P>.05). | **effectiveness**: SUD, STAI tx-1, burnout scale |
| Graziano (2021)[38] | Italy | March to May 2020 | individuals with cystic fibrosis and their caregivers | to evaluate the effectiveness of a cognitive‐behavioral telehealth intervention to reduce symptoms of stress, depression, and anxiety in pwCF and parents during the lockdown. | psychotherapy; CBT | The 4‐session intervention focused on self‐care, coping skills, exercises to improve mood, and individual, emotional challenges. CBT skills included: cognitive reframing (facilitating positive thoughts), relaxation training, increasing positive emotions, and getting physical exercise; 30–40 min each, conducted by a clinical psychologist on the CF Team. | **feasibility** and **acceptability**: generally high ratings. **effectiveness**: Ratings of stress significantly decreased from pre‐ to post‐testing for pwCF (P<.01) and parents (P<.001). Significant reductions in depression for pwCF were found (P<.05) but not anxiety (P=NS). Parental depression decreased for parents (P<.05), but not anxiety (P=NS). | **feasibility**: two items;  **acceptability**: two items on satisfaction  **effectiveness**:  perceived ratings; depressive symptoms: PHQ‐8, anxiety: GAD‐7 |
| Gromatsky (2021)[39] | US | not specified | veterans experiencing acute stress related to COVID-19 | piloted a novel telehealth group-based intervention for Veterans experiencing acute stress related to COVID-19, titled VA Caring for Our Nation’s Needs Electronically during the COVID-19 Transition (VA CONNECT), and examined feasibility and acceptability | psychotherapy; cognitive behavior therapy, DBT skills, psychoeducation, and peer support to promote effective coping, emotion management, and stress relief pertaining to COVID-19 distress | VA CONNECT is a 10-session group delivered twice weekly followed by four optional, weekly sessions. VA CONNECT integrates elements of cognitive behavior therapy, DBT skills, psycho-education, and peer support to promote effective coping, emotion management, and stress relief pertaining to COVID-19 distress. | **feasibility**: all referred veterans expressed interest in VA CONNECT and consented; attendance and retention were also strong. **acceptability**: participants' perceptions of intervention acceptability, appropriateness, and feasibility were very high; a third of the participants reported subjective stress and/or symptom reduction. A majority (75%) noted participation increased interpersonal connection and reduced isolation | **feasibility**: rate of successful referral (recruitment) and rate of overall and session-specific attendance (retention); **acceptability**: Acceptability of Intervention Measure |
| Karagiozi (2021)[48] | Greece | not specified | caregivers of patients with dementia | to assess the benefit from the effects of the online versus onsite psychoeducative interventions on caregivers’ emotional burden | psychoeducation; psychoeducation interventions | 4-month intervention period; 16 sessions for 60 minutes per session which include 1) general information about dementia, 2) common challenges of caregiving- difficulties faced by caregivers, 3) problems and difficulties faced by care receivers 4) Ways of coping with behavioral disturbances | **effectiveness**: No significant differences were found between the online and onsite groups in anxiety (P=.531) and depression (P=.577) after the interventions. Both interventions showed significant reductions across emotional variables measured over the course of the treatment study and treatment interventions | **effectiveness**: BAI, BDI |
| Lazzaroni (2021)[51] | Italy | February to May 2020 | adolescents and young adults (aged between 13 and 24 years) | to measure changes in the levels of traumatic stress and anxiety in a clinical population of adolescents and young adults aged 13 to 24 years, following a trauma-focused psychotherapeutic group intervention according to the eye movement desensitization and reprocessing protocol, conducted remotely before the end of the first lockdown | psychotherapy; eye movement desensitization and reprocessing | participants were divided into small groups of three or four people; participated in three group meetings of 1 h each, delivered online according to the brief EMDR group treatment protocol created by the re-elaboration of the guidelines for the stabilization-decompression of Critical Incident Stress Management and the specific EMDR protocols for Acute and Recent Traumatic Events. | **effectiveness**: a significant improvement pre- and post-intervention in the scores of the scales STAI, IES-R, and Emotion Thermometer with a reduction in post-traumatic symptoms related to the domains of intrusiveness and hyperarousal | **effectiveness**: IES-R; STAI; Emotion Thermometer |
| Maldonado (2020)[53] | US | August to September 2020 | adults (aged ≥18 years) | to determine if a six-week online mental health promotion intervention would improve the GAD-7 scores of participants. | psychoeducation; multiple-components: a mental wellness program that showed how healthy lifestyle activities such as physical activity, mindfulness, sleep, social connectedness, and nutrition may decrease the impact of anxiety disorder | a six-week online mental health promotion intervention; participants watched the online mental health program Sane through COVID with WILD 5-Wellness KickStart30 introductory video after completing the pretest; then received a link to the video presentations weekly for the next five weeks via email provided | **effectiveness**: a statistically significant increase from the mean pre-test score to post-test score following the intervention (P<.05) | **effectiveness**: GAD-7 |
| Sharrock (2021)[63] | Australia | 12th September 2019 to 11th March 2020 and 12th March to 11th June 2020 | adults | to examine the uptake and outcomes of the THIS WAY UP iCBT for Health Anxiety course in an open evaluation during the early COVID-19 pandemic period in Australia | psychotherapy; CBT | Participants are given 90 days to complete the program. The Health Anxiety program consists of six comic-based online lessons following the story of a fictional character who experiences health anxiety and learns CBT skills with the help of a clinician. After each lesson, the participant downloads the lesson homework which includes practical exercises. | **usability**: significant increases in course registrations; and course commencements; **feasibility**: course completion during COVID was 30.5%; **effectiveness**: significant improvements in health anxiety (g=0.89), and distress (g=0.91), and medium improvements in depression (g=0.55) were found | **usability**: number of monthly course registrations and course commencements; **feasibility**: course completion; **effectiveness**: Short Health Anxiety Inventory, PHQ-9, and Kessler-10 |
| Sun (2021)[69] | China | March to June 2020 | university students | to examine the effectiveness of a mindfulness-based mHealth intervention in reducing symptoms of anxiety and depression for young adults in quarantine compared to a rigorous active control (social support mHealth) | psychotherapy; Mindfulness-Based Stress Reduction and Mindfulness-Based Cognitive Therapy | The Mindfulness for Growth and Resilience is a 4-week intervention; Zoom was used to provide weekly one-hour meetings for experiential and group learning of mindfulness; a WeChat-based miniprogram was developed for (a) didactic learning regarding mindfulness (3 min each, 5 videos per week) and (b) audio-based daily practice (2 per week, which varied from 5 to 40 min each); a messaging-based group via WeChat was used for asynchronous group communication and discussion | **feasibility**: participants in the mindfulness mHealth condition had high attendance in weekly videoconferencing sessions and high retention rates (91.2%); **acceptability**: mindfulness group reported significantly higher ratings across most acceptability indicators; the difference in self-reported adverse effects was nonsignificant (3.9% in mindfulness and 8.7% in social support); **effectiveness**: compared to social support mHealth, mindfulness mHealth had a superior effect on anxiety (P=.024, d=0.72), both conditions improved on depression | **feasibility**: attendance and retention rates; **acceptability**: structured surveys and open-ended questions; **effectiveness**: GAD-7, PHQ-9, Mindful Attention Awareness Scale, Multidimensional Scale of Perceived Social Support |
| Tarquinio (2021)[70] | France | March to May 2020 | health professionals | to show how the use of the URG-EMDR protocol in a telemental health setting proved feasible and effective in the treatment of a group of healthcare professionals working in nursing homes or hospitals during the acute phase of the COVID-19 pandemic | psychotherapy; Eye Movement Desensitization and Reprocessing therapy | the URG-EMDR treatment is a single-session treatment with 8 phases; the average duration of the URG-EMDR treatment (phases 3 to 7) was 2 hours and 14 minutes (66% of sessions were completed in less than 2 hours) | **feasibility**: the remote therapy setting was judged satisfactory by the patients, even if it required adjustments and certain recommendations for practice;  **effectiveness**: significant differences between pre-test and post-test after 24 hours, which is consistent with a decrease in scores on anxiety, depression, and perceived disturbance; the result is maintained 1 week after the intervention | **feasibility**: six questions; **effectiveness**: HADS Subjective Units of Disturbance, fear of going to work and fear for your safety at work (two questions) |
| Vallefuoco (2021)[71] | Italy | not specified | therapists and parents of children with ASD | to provide an overview of the SUPER platform’s structure and report the results from a preliminary usability test | psychiatry rehabilitation; ASD International Guidelines and the recommendations of the International Classification of Functioning, Disability, and Health for Children and Youth | SUPER is a digital platform that provided two main areas; the General Area provided science-based information on ASD, the Perssonalized Area provided useful tools for evaluation and monitoring in different life contexts; the researchers provided a short virtual tour of SUPER, describing the platform's structure and tools for 10 min, then participants were asked to access SUPER and use it for at least 30 min, but not more than 120 min, and to perform a set of tasks | **usability**: The total mean SUS score (89.2) showed that SUPER is an excellent and usable system | **usability**: system usability scale |
| Wahlund (2020)[74] | Sweden | May to July 2020 | adults who self-identified as being excessively worried about the COVID-19 pandemic and its consequences | to investigate if a brief self-guided, online psychological intervention can reduce the degree of dysfunctional worry related to the COVID-19 pandemic and associated symptoms | psychotherapy; cognitive behavioural interventions for worry-related problems | 3-week, self-guided program, provided via a secure, encrypted study website and organised in five brief modules: worry and the evolutionary function of worrisome thoughts, problem-solving techniques, identify, register, and refrain from any un- helpful checking or reassurance-seeking behaviours, detaching oneself from un- helpful worrisome thoughts, engage in competing focus-shifting behaviours | **feasibility**: 201 of 335 (60%) participants completed at least three modules and 123 (37%) completed all five modules; 35 self-reported adverse events; **acceptability**: high satisfaction;  **effectiveness**: significant reductions in COVID-19-related worry with a medium effect size (d=0.74 [95% CI: 0.58–0.90]); Improvements on mood, daily functioning, insomnia, and intolerance of uncertainty | **feasibility**: completed rate **acceptability**: an adapted version of the Client Satisfaction Questionnaire. **effectiveness**: GAD-7; Work and Social Adjustment Scale, Montgomery Åsberg Depression Rating Scale–Self rated, Intolerance of Uncertainty Scale short version, ISI |
| Wasil (2021)[75] | US | March to April 2020 | graduate and professional students | to evaluate the acceptability and perceived utility of COMET during COVID-19 | psychotherapy; behavioral activation, cognitive restructuring, and gratitude | participants were randomly assigned to receive two of the three intervention modules; self-administered digital single-session intervention | **feasibility**: 189 individuals completed (72%).  **acceptability**: the intervention modules were acceptable (93% endorsing), helpful (88%), engaging (86%), applicable (87%), and could help manage COVID-related challenges (88%). **effectiveness**: significant improvements in secondary control and in the perceived negative impact of the COVID-19 crisis on their quality of life (P<.001） | **feasibility**: completed rate;  **acceptability**: Acceptability of Intervention Measure;  **effectiveness**: secondary control scale; self-developed questions relating to the COVID-19 pandemic |
| Wei (2020)[17] | China | Feb 2 to Feb 28, 2020 | COVID-19 patients | to introduce an Internet-based integrated intervention to COVID-19 patients with psychological distress and to evaluate the efficacy of the provided mental health care on depression and anxiety symptoms in patients with COVID-19 | psychotherapy; four main components: breath relaxation, mindfulness, "refuge" skills, and the butterfly hug method | a self-help intervention; patients listened to audio-recorded instructions via their mobile phones; for 50 mins every day for two weeks | **effectiveness**: decreased levels of depression and anxiety symptoms (P<.05) | **effectiveness**: depression and anxiety symptoms: 17-HAMD, and HAMA, respectively |
| Wood (2021)[76] | US | March to June 2020 | individuals with a diagnosis of a primary psychotic disorder | to implement group teletherapy for two small cohorts of individuals with FEP receiving care in a coordinated specialty care clinic using methods adopted from Acceptance and Commitment Therapy | coordinated specialty care; acceptance and commitment therapy | group teletherapy hosted using secure software, led by a licensed clinical psychologist, aided by a peer support specialist; 4 sessions included values, unusual experiences/anxiety, relationships, life goals; 75 minutes per session | **acceptability**: high adherence with group visits as well as client satisfaction across groups | **acceptability**: CSQ, feedback questionnaire |
| Ying (2021)[77] | China | January to March 2020 | older adults | to explore the feasibility and effectiveness of ICBT intervention among older adults living in nursing homes in China during the COVID-19 pandemic | psychotherapy; cognitive behavioral therapy | a 5-week long clinician-guided ICBT delivered through a WeChat mini-program | **usability**: the mean total clinical psychologist time spent with each participant for the entire program was 35.63 min (SD = 25.41);  **feasibility**: 87.4% (111/127) participants completed in 5 weeks; 4.74 (SD = 0.81) lessons completed **acceptability**: a high level of satisfaction **effectiveness**: improvements in depressive symptoms, anxiety symptoms (P<.001) and were sustained at 1-month follow-up | **usability**: time engagement;  completed rate, **acceptability**: two self-administrated questions;  **effectiveness**: depressive symptoms: PHQ-9 and GDS, anxiety symptoms: GAD-7; general psychological distress: K-10 |
| Zepeda (2021)[78] | Canada | April to September 2020 | children | to investigate the feasibility, acceptability, and preliminary effectiveness of iCOPE with COVID-19 | psychoeducation; CBT and DBT | three weekly sessions: (1) building rapport with the client, providing psychoeducation about COVID-19 and emotions, and practicing a relaxation strategy to manage anxiety; (2) reviewing homework from Session 1 and identifying other strategies for emotion regulation, (3) helping children develop strategies to manage emotions by using mindfulness, present-moment strategies, and dialectical behavioral strategies | **feasibility**: only 1 child did not complete all three sessions of the protocol and 4 received four sessions, and no technological difficulties were reported; **acceptability**: an overall positive response to the protocol; **effectiveness**: significant reductions in social anxiety | **feasibility**: successful referral, retention of participants to the treatment protocol, and technical failures  **acceptability**: satisfaction questionnaires;  **effectiveness**: anxiety symptoms: Screen for Child Anxiety and Related Disorders |
| Zimmerman (2021)[80] | US | May 2020 to October 2020 | psychiatric patients | to compare the satisfaction of partial hospital programs services delivered via telehealth to in-person treatment provided to patients treated prior to the COVID-19 outbreak | psychiatric visits and psychotherapy; Rhode Island Methods to Improve Diagnostic Assessment and Services including assessments, individual therapy, psychiatry visits, and group therapy sessions | regular psychiatry sessions; daily individual therapy sessions of approximately 30 to 45 min; 3 daily therapy groups; an optional mindful mediation group | **feasibility**: 75.0% (180/240) completed the course of treatment (n = 60); **acceptability**: 90% of the patients (118/131) were very or extremely satisfied with the initial evaluation; 93.2% were very or extremely satisfied with their treatment; lesser satisfaction with the interpersonal process group; 95.6% would recommend it to others | **feasibility**: completed rate;  **acceptability**: Clinically Useful Patient Satisfaction Scale |
| ***Smartphone applications*** | | | | | | | | |
| Al-Refae (2021)[22] | Canada | August 29 to December 6, 2020 | adults | to test the efficacy of a wellness smartphone application (Serene) on measures of stress, depression, anxiety, self-compassion, well-being, and wisdom | psychotherapy; mindfulness and self-compassion practices | Participants were instructed to do at least one mindfulness meditation a day of their choice during the period of 30 days. They were also asked to do a cognitive restructuring task, as needed. No minimum or maximum amount of cognitive restructuring tasks were required. Depending on each participant, they may choose to perform one task (i.e., they may continue to modify one plan to address one situation) or engage in more than one task throughout the study. A journaling section was available for use but was not required for participants to engage in. | **usability**: participants in the intervention group engaged in 5 meditations a week. **effectiveness**: significant moderate between-group differences for depressive symptoms (d=-0.43) and decisiveness (d=0.34). Moderate between-group differences were also found for self-compassion (d = 0.6) such that significant improvements in self-kindness, common humanity, mindfulness, and decreases in self-judgement, isolation, and overidentification were observed. A small between-group difference was found for emotional regulation (d=0.28). A significant moderate within-group decrease in stress (d=-0.52) and anxiety symptoms (d=-0.47) was also observed in the intervention group | **usability**: engagement; **effectiveness**: DASS-21, San-Diego Wisdom scale, Abbreviated Three-Dimensional Wisdom scale, Psychological Well-being scale; SCS |
| Ben-Zeev (2021)[24] | US | recruited from January to September 2020 | people with serious mental illness | to evaluate the clinical effectiveness of a smartphone app (CORE) designed to challenge dysfunctional thoughts that underlie common symptoms of SMI, self-stigmatizing attitudes, and maladaptive beliefs that impede treatment seeking and recovery | psychoeducation; psychiatric recovery; produce changes in the relative activation of adaptive and maladaptive beliefs about the self, others, and the world such that adaptive beliefs would be more easily retrieved than maladaptive ones | participants were given access to the CORE app for 30 days of use. After a month, they concluded the intervention and uninstalled the app. The intervention comprises daily brief game-like exercises. CORE users were trained to respond to multiple statements in a sequence of modules that progress through the following domains: self-talk, belief in change, self-stigma, self-care, self-worth, illness and identity, personal strength, social avoidance, feelings versus facts, catastrophization, thoughts of reference, paranoid ideation, treatment seeking, and recovery | **acceptability**: highly usable and acceptable; **effectiveness**: significant treatment time interactions for the BDI-II (P<.001), GAD-7 (P=.01), RAS (P<.001), RSES (P<.001), and SDS (P=.001). Large effects were observed for the BDI-II (d=0.58), RAS (d=0.61), and RSES (d=0.64); moderate effect size was observed for the SDS (d=0.44), and small effect size was observed for the GAD-7 (d=0.20). Similar changes in outcome measures were later observed in the waitlist control group participants following crossover after they received CORE (T2 to T3) | **acceptability**: a self-report usability and acceptability measure; **effectiveness**: BDI-II, GAD-7, Program for Schizophrenia Voices  Questionnaire, Program for Schizophrenia Voices  Questionnaire, Recovery  Assessment Scale, RSES, Friendship Scale, SDS |
| Chandra (2021)[27] | India | March 21 and June 25, 2020 | COVID-19 Suspect and Positive Patients in Isolation Facilities | to evaluate the acceptability of mHental Health Support Counselling Service | mental health support counselling; supportive psychotherapy, psychoeducation, counselling regarding testing protocol, sleep hygiene, relaxation techniques, activity scheduling, etc. | Tele counsellors assessed the allocated patients telephonically and provided appropriate psychosocial interventions like Supportive Psychotherapy, Sleep Hygiene, Grief Counselling, etc. Any issue that they could not address themselves was posted in a WhatsApp group for first level advice by the core team and second level guidance by Chief Supervisor. The status of these cases was updated on WhatsApp till transfer, discharge or death | **acceptability**: most respondents expressed satisfaction on pilot anonymised feedback with 83% recommending the programme. | **acceptability**: client Satisfaction Scale |
| Coifman (2021)[28] | US | May and June 2020 | medical and emergency personnel | to test the efficacy of a brief and novel online ambulatory intervention aimed at supporting psychological health and well-being for medical personnel and first responders during the COVID-19 pandemic | psychotherapy; expressive writing, adaptive emotion regulation activity, and positive emotion-generation activities | The once-a-day (3–6min) intervention included three steps: (1) typing a narrative of challenges occurring that day, which constituted an expressive writing activity; (2) practicing adaptive emotion regulation by revisiting distressing events from a distanced perspective, which constituted an explicit self-distancing activity; and (3) responding to one or two, depending on dose, (of eight) randomised prompts to generate PE. | **acceptability**: ratings from the 12 participants (43%) who responded to follow-up suggested that acceptability and perceived efficacy were good: 70% rated the intervention as having moderate to high effectiveness and 70% rated negative side effects as unlikely. **effectiveness**: the results indicated a 13% increase in positive emotion, P=.056; and decrease in negative emotion by 44%, P=.001 across both doses. | **acceptability**: single-item ratings； **effectiveness**: daily emotion ratings |
| Esenturk (2021)[33] | Japan | March 30 to April 26, 2020 | children with ASD and their parents | evaluate the feasibility of WhatsApp-based physical activities to increase the physical activity level of children with ASD during the COVID-19 pandemic | psychiatry rehabilitation; physical activity period, home-based exercises, fun games, daily housework, dance, meditation, and fitness activities | physical activity sessions were conducted for 4 weeks, 7 days/week for 20–30 min. Activities that parents could easily implement at home with their children with ASD and that did not require any materials were included in the sessions. Each physical activity session consisted of (a) a warm-up period of about 10 min, (b) a physical activity period of about 10 min, and (c) a stretching cooldown period of about 10 min. The warm-up period included mild gait and rotation of the joints in the extremities (knee and ankle, hip, neck, and shoulder). Once per week, a chat was carried out with the parents about at what rate the physical activities were performed | **feasibility**: the parents (85.7%) stated that the physical activity contents shared in the group via WhatsApp were useful during the quarantine process due to the COVID-19 pandemic. Most parents (64.2%) found it very useful to include themselves in WhatsApp-based physical activities. **effectiveness**: When the pretest to posttest physical activity levels of children were compared, a statistically significant difference was observed in favor of the posttest (P<.05) | **feasibility**: feasibility questionnaire. **effectiveness**: LTEQ |
| Fiol-DeRoque (2021)[34] | Spain | May 14 to July 25, 2020 | health care workers aged >18 years who had provided health care to patients with COVID-19 | to evaluate the effectiveness of PsyCovidApp (a self-managed and self-guided psychoeducational mobile-based intervention with no therapist support) to reduce symptoms of depression, anxiety, stress and other mental health problems in health care workers during the COVID-19 pandemic in Spain | psychotherapy; CBT and mindfulness | targeting four areas: emotional skills, healthy lifestyle behavior, work stress and burnout, and social support; the intervention included daily prompts that included brief questionnaires to monitor mental health status, followed by short messages offering tailored information and resources based on the participants´ responses | **usability**: The mean usability score of PsyCovidApp was high (87.21/100, SD 12.65). **acceptability**: After the trial, 208/221 participants in the intervention group (94.1%) were asked to regain access to PsyCovidApp. **effectiveness**: observed significant improvements among health care workers consuming psychotropic medications in the primary outcome (P=.004), and in posttraumatic stress, insomnia, anxiety, and stress. Similarly, among health care workers receiving psychotherapy, we observed improvements in the primary outcome (P=.02), and in insomnia, anxiety, and stress. | **usability**: system usability scale; **acceptability**: the number of the participants asked to regain access to PsyCovidApp. **effectiveness**: DASS-21; Davidson Trauma Scale;; MBI-HSS; ISI; GSE |
| Gordon (2021)[37] | US | May 1, 2020, to December 31, 2020 | general population | to develop the See Me Serene app and test the feasibility and acceptability of study procedures, and explore the potential impact of the app on stress and anxiety | psychotherapy; guided imagery | The See Me Serene app allows users to choose from a variety of guided imagery audio files and associated photos of immersive, evocative nature scenes. Users can select audio files from guided imagery categories. Each file contains detailed, vivid descriptions of the scenarios, including sights, sounds, smells, tastes, tactile sensations, and emotions. Each audio file starts with a brief relaxing breathing exercise and instructions to “release any tension in your body and mind.” Users receive notifications to listen to the files once each day. See Me Serene tracks the user’s mood each time the user logs in with 4 questions assessing how often/much the user has felt stressed, anxious, lonely, or worried “today”. | **feasibility**: 85% (84/99) retention rate; participants listened to 48.2 audio files (range 0-280) over 30 days, or approximately 1.6 audio files per day. **acceptability**: Participants were satisfied with the app, with 87% (78/90) rating the app as helpful in dealing with stress and anxiety.  **effectiveness**: may have the potential to reduce anxiety, participants reported reductions in self-reported stress (P=.001), symptoms of PTSD (P<.001), anxiety (P<.001), loneliness (P<.001), and worry (P=.007) from pre- to posttest. | **feasibility**: participant retention and adherence; **acceptability**: satisfaction; **effectiveness**: PSS; Overall Anxiety Severity and Impairment Scale; IES-R; UCLA; Penn State Worry Questionnaire |
| Hosseinzadeh Asl (2021)[43] | Turkey | not specified | social workers | to investigate whether a brief mindfulness-based intervention had short- and middle-term effects on social workers working during the COVID-19 pandemic. | psychotherapy; mindfulness | The intervention consisted of four weekly 70-min mindfulness training sessions plus 10 to 20 min of daily meditation as homework. Each session was divided into two parts lasting approximately 35 min, with 10 min of rest time between. The sessions typically began with mindfulness-based psychoeducational materials and followed by meditation exercises. | **effectiveness**: statistically significant improved in psychological flexibility (P<.01), selfcompassion (P<.01) and depression (P<.01) | **effectiveness**: DASS-21; Self-Compassion Scale; Acceptance and Action Questionnaire-II |
| Jaworski (2021)[46] | US | May 1 to October 31, 2020 | app user | to describe the general usage trends and patterns of COVID Coach | psychoeducation; coping tools, psychoeducation, self-assessments, and accessing resources | COVID Coach, available for Android and iOS, was designed for the COVID-19 pandemic to provide users with interactive, evidence-informed tools for coping with stress and anxiety, information about how to stay well, stay connected, and navigate challenges, self-monitoring mental health symptoms and goals, and resources to discover and connect with various types of verified and vetted support | **usability**: daily active users range from 1205.77 (SD=615.7) to 611.35 (128.6); monthly active usage has remained steady over time (11,000 users/month); **feasibility**: 86.8% users had their first day of app use; 51.4% used the app for more than 2 days; the number of days retained was 42.44 (SD=44.40) | **usability**: daily active users, monthly active usage; **feasibility**: the number of days retained, and the proportion of uses retained |
| Parolin (2021)[57] | Italy | September to November 2020 | community population | to describe an app-based group psychological intervention supporting individuals experiencing psychological distress during the 2020 pandemic in the Lombardy region in Italy | psychotherapy; emotion regulation strategies | The ‘Italia Ti Ascolto’ provides different online rooms, themed according to a specific target population and guided by a licensed group psychotherapist; consisted of 16 up to 20 rooms per week for 3 months; Each room lasted one hour and hosted up to 16 participants | **usability**: overall user experience scored 88.98 (SD=13.56) and it is categorized as 'Best possible' | **usability**: system usability scale |
| Philip (2020)[58] | France | April 22 to May 5, 2020 | adults | to assess the feasibility of KANOPEE, in terms of inclusion rate, follow-up rate, perceived trust and acceptance of the virtual agent, and effects of the intervention program, in the context of COVID-19 confinement in France | psychological assessment and psychotherapy; sleep assessments and personalized sleep interventions | The users first completed a screening interview based on the ISI conducted by the virtual agent. If the users were positive for insomnia complaints (ISI score >14), they were eligible to join the 2-stage intervention program: (1) complete an electronic sleep diary for 1 week and (2) follow personalized sleep recommendations for 10 days | **feasibility**: Of the 773 screened users who reported sleep complaints (ISI score >14), 166 (21.5%) followed Step 1 of the intervention, and only 47 of those (28.3%) followed Step 2;  **acceptability**: 76% (1574/2069) of the app users completed the screening interview with the virtual agent. The virtual agent was well accepted by 27.4% (431/1574) of the users; **effectiveness**: insomnia complaints and nocturnal sleep quality improved significantly after 1 week (P<.005). Users who completed Step 2 also showed an improvement compared to the initial measures (P<.001). | **feasibility**: drop-out rates;  **acceptability**: Acceptance and Trust Questionnaires; **effectiveness**: ISI and nocturnal sleep indicators |
| Song (2021)[64] | China | April to June 2020 | adult residents who had depression symptoms | to explore the intervention efficacy of a mobile application on addressing psychological problems related to COVID-19 | psychotherapy; CBT | The “Care for Your Mental Health and Sleep during COVID-19” (CMSC) is a self-help storytelling application based on CBT theory. It comprises three parts: evaluation, psychological intervention, and other (courses to improve sleep quality, relaxation training, and national psychological aid hotline). Each session had four different stories that took about 30 min to complete. | **acceptability**: CMSC was considered helpful (n=68, 81.9 %) and enjoyable (n=54, 65.9 %) in relieving depression and insomnia during the COVID-19 outbreak; none reported adverse events. **effectiveness**: significant improvement in depression and insomnia (P<0.05) compared with the wait-list group | **acceptability**: visual analogue scale on satisfaction;  **effectiveness**: PHQ-9; GAD-7, visual analogue scale; ISI; the Chinese version of the Connor Davidson resilience scale |
| Sturgill (2021)[66] | US | launched at the end of January 2020 | college students | to determine the effectiveness of delivering an EI curriculum and mindfulness techniques using an AI conversation platform, Ajivar, to improve symptoms of anxiety and depression during this pandemic | psychoeducational; psychotherapy; mindfulness, positive psychology, resiliency, and emotional intelligence training | Ajivar delivers a personalized emotional intelligence curriculum and mindfulness techniques; components included psychoeducational content of positive psychology; mindfulness; personalization; resiliency and emotional intelligence training; reflection; positive affirmations; engagement. | **usability**: interacted with Ajivar for a mean time of 1424 (SD 1168) minutes; **effectiveness**: a significant decrease in anxiety and the symptoms of depression measured at the start of the study compared to the end (P=.001) | **usability**: User Engagement and Attrition**;**  **effectiveness**: The TestWell Wellness Inventory; GAD-7; PHQ-9; Emotional Quotient |
| Summers (2021)[68] | UK | February 26 to March 27, 2020 | adults | to evaluate the 12-week outcomes of the digitally delivered Gro Health intervention | mental well-being support; education around minimizing the risk of infection and spread of COVID-19; mindfulness, mental well-being; meditation | Gro Health app is a digital health intervention that provides 13 modules in response to COVID-19 and digital tools for self-monitoring data activity (eg, steps and distance), body weight, blood pressure, heart rate, mood, food intake, body weight, and blood glucose levels; provided weekly automated feedback and notifications weekly to engage within the app; 12-week intervention | **usability**: the mean number of engaged minutes with the well-being function of the Gro Health app was 36.74 (SD 25.9) minutes, **effectiveness**: statistically significant changes in scores for anxiety, perceived stress, and depression (P<.001) | **usability**: total minutes of app engagement; **effectiveness**: GAD-7, PHQ-9, Perceived Stress Scale |
| ***Text messaging*** | | | | | | | | |
| Aguilera (2021)[20] | US | April to December 2020 | adults | to assess the effects of StayWell on symptoms of depression and anxiety in a broad adult population during the COVID-19 pandemic | psychotherapy; CBT | 2 messages daily for 60 days: 1 skills-based message and 1 message inquiring about their mood. The skills-based messages included tips on how to deal with worry and stress brought on by the COVID-19 pandemic. Messages were sent daily at a random time between 9 AM and 6 PM. | **effectiveness**: significant reductions in both PHQ-8 and GAD-2 scores from baseline. We found an average reduction of -1.72 (95% CI -2.35 to -1.09) in PHQ-8 scores and –0.48 (95% CI -0.71 to -0.25) in GAD-2 scores. These improvements translated to an 18.5% and 17.2% reduction in mean PHQ-8 and GAD-2 scores, respectively | **effectiveness**: GAD-2; PHQ-8 |
| Agyapong (2021)[21] | Canada | April 26 to July 12 2020 | general population | to assess the effectiveness of Text4Hope in reducing psychological impacts due to COVID-19 | psychotherapy; CBT | a different non-personalized pre-programmed message from a web application each day for six weeks. Messages were designed in the framework of CBT. | **effectiveness**: participants in the intervention group had lower prevalence rates for moderate/high stress, likely GAD, and likely MDD, compared to respondents in the control group. After controlling for demographic variables, the IG remained less likely to self-report symptoms of moderate/high stress, likely GAD (OR = 0.55; 95% CI = 0.44–0.68), and likely MDD (OR = 0.50; 95% CI = 0.47–0.73). The mean Composite Mental Health score, the sum of mean scores on the PSS, GAD-7, and PHQ-9 was 20.9% higher in the control group | **effectiveness**: PSS, GAD-7, and PHQ-9 |
| Ortiz (2021)[56] | US | April 13 to May 8, 2020 | adults aged >18 years; discharged from two emergency departments | to investigate the ability of a health system–based digital, remote, interactive tool to provide health and well-being resources to local community participants and to foster connectivity among them during the early phases of COVID-19 | psychological assessments; mood management | a platform (Mosio) was used to communicate with them through two-way texting for 4 weeks; received weekly text messages asking them to rate their mood; Tiers of mood rating by the participants trigger the receipt of differential content. Participants reporting a “high” mood rating of 7-10 would always receive a link to an uplifting resource, whereas those reporting a “low” mood rating of 0-3 always received links to mental health and well-being resources. Those reporting a medium” mood rating of 4-6 received links to either an uplifting resource or to the resource homepage to view all featured resources, alternating each week. | **feasibility**: 236 (83%) responded with a mood rating at week 1, and 175 (60%) responded during week 1 and to the follow-up text during week 4, including 101 (35%) who responded during weeks 1 and 4. Furthermore, 45 (16%) participants responded during all 4 weeks; **effectiveness**: mood improved significantly among participants who reported a low mood rating at baseline (P<.05) | **feasibility**: follow-up rate; **effectiveness**: mood rating, qualitative description |
| Shalaby (2021)[61] | Canada | May to July 2020 | community population | to evaluate the feedback, satisfaction, experience, and perceptions of Text4Hope subscribers | mental well-being support; in line with a cognitive behavioral framework | Individuals self-subscribed to Text4Hope to receive daily supportive text messages for 3 months; Text message delivery was unidirectional and not specifically tailored to the end-users; The messages were uploaded to a web-based platform, which delivered automated messages at 9 AM | **acceptability**: the mean overall satisfaction score was 8.55 (SD 1.78), suggesting high overall satisfaction with Text4Hope; most subscribers agreed that Text4Hope helped them cope with stress and anxiety, feel connected to a support system, manage COVID-19–related issues, and improve mental well-being; subscribers agreed that messages helped them cope with stress, were positive, affirmative, and succinct | **acceptability**: Text4Mood user satisfaction survey; the perceived impacts,  subscribers' feedback survey |
| Suffoletto (2021)[67] | US | August to October 2020 | youths (18 years or older) with a current mental health diagnosis | to test the acceptability and effects of an automated digital Mobile Support Tool for Mental Health (MoST-MH) for young adults transitioning to college | psychological assessments; ecological momentary intervention | incorporated periodic text message mental health check-ins, triggering web-based check-ins (when mental health was rated low) to understand stressors, negative effects, and self-efficacy, which informed self-efficacy support strategies and prompted links to psychoeducational videos focused on college and mental health; 3 months in total | **usability**: high usability;  **feasibility**: MoST-MH participants were sent a median of 5 (range 3 to 10) text message check-in prompts over the 3-month study period and 100% were completed; participants were sent a median of 2 (range 1 to 8) web-based check-in prompts among which 78% (43/55) were completed. **effectiveness**: reductions in mental health symptoms over time and significant between-group effects of MoST-MH compared to enhanced usual care on depressive symptom severity. No significant differences in mental health self-efficacy or mental health care use were observed. | **usability**: Post-Study System Usability Scale; **feasibility**: text messaging and web-based responses; **effectiveness**: Mental Health Self-Efficacy Scale, College Counseling Center Assessment of Psychological Symptoms, Client Service Receipt Inventory for Mental Health |
| ***Social media*** | | | | | | | | |
| Hu (2021) [44] | China | February 2, 2020, to February 29, 2020 | patients with suspected COVID-19 | Investigated the effects of a new WeChat-based psychological intervention on the mental health of suspected NCP patients. | psychotherapy; comprehensive components: psychological counseling/Health education/relaxation therapy | twice-daily psychological counseling delivered via WeChat video communication; Provide daily updates regarding the epidemic and related policies; Once-daily relaxation therapy, and whole-body relaxation training via WeChat phone sessions. Offered personalized WeChat tips, including medication regimens, work and rest, meal times and diet, self-protection, and appropriate in-room exercise; lasted 14 days | **effectiveness:** the intervention group showed an improvement in anxiety (P<.05) and a significant improvement in depression and acute stress disorder compared with the control group (P<.01). | **effectiveness**: SAS,  SDS, ASDS |
| McKeon (2021)[54] | Australia | June to August 2020 | people over the age of 60 years | to assess the feasibility and preliminary effectiveness of a 6-week mental health-informed lifestyle program, delivered to older adults via Facebook during the second peak of COVID-19 cases | mental well-being support; goal setting, balance training, reducing sedentary behaviour and diet | a 6-week lifestyle intervention delivered via a private Facebook group with the monitor by the facilitators; weekly content was posted in the form of information, exercise demonstration videos, links to existing online resources, and discussion questions; participants can join a weekly 30- to 45- min group video call via Zoom, and can chat, ask questions, and socialise with other users | **feasibility**: all 11 (100%) remained in the private Facebook group; five participants engaged in the group video calls;  **acceptability**: high acceptability was observed; **effectiveness**: evidence of an effect on psychological distress, quality of life, functioning, loneliness and physical activity | **feasibility**: the usage of the Facebook group; participant retention; **acceptability**: 14-item feasibility and acceptability questionnaire.  **effectiveness**: K10, AQoL-6D, UCLA loneliness scale, Suicidal Ideation Attributes Scale |
| Zhang (2021) [79] | China | January 2020 to March 2020 | residents meet the baseline threshold score of the BSI-18 | to evaluate a mindfulness-based intervention for psychological distress among Chinese residents during COVID-19 | psychotherapy; mindfulness | the intervention group received 2h psychoeducation sessions and 1.5 h of practice per day for 13 days | **usability**: 18.68 (SD: 4.71) hours for 13 days; **effectiveness**: significant improvement in mindfulness awareness, psychological distress, somatic symptoms, depressive symptoms, and anxiety symptoms (P<0.001) | **usability**: daily mindfulness activities;  **effectiveness**: BSI-18, Mindful Attention Awareness Scale |
| ***Hotline & telephone calls*** | | | | | | | | |
| Geoffroy (2020)[36] | France | March 18 to April 12, 2020 | hospital workers | to present the methods for implementing such psychosocial support system (Covid-Psy hotline) and characterize first calls and reasons for the call. | psychotherapy; no specific crisis intervention models or algorithms were used | the COVID-Psy hotline time was composed of certified psychologist volunteers, with the capacity of three numbers to respond to all calls. Assistance including the reason for the call, identification of symptoms, proposal of responses according to guidelines and to symptoms identified, short individual response, referral to other psychosocial support, and medical specialized additional expertise | **usability**: 149 calls within 26 days (5.73 calls/day, SD=3.22); 18.5 min/call, SD=14.7 | **usability**: daily activities, duration of a call |
| Kahlon (2021)[47] | US | July 6 to September 24, 2020 | older adults | to determine whether a layperson-delivered, empathy-focused program of telephone calls could rapidly improve loneliness, depression, and anxiety in at-risk adults | mental well-being support; empathetic conversations | All participants were called 5 days during the first week. After this, participants chose the frequency of calls (2-5 calls/week). Calls were targeted to be less than 10 minutes but there were no time limits with participants. | **feasibility**: 9/120 dropped out; **effectiveness**: improvements in loneliness, depression, anxiety, and general mental health (P<0.05) | **feasibility**: drop-out rates;  **effectiveness**: UCLA Loneliness Scale; De Jong Giervald Loneliness; PHQ-9; GAD-7; Short Form Health Survey Questionnaire |
| Sosa Lovera (2021)[65] | Dominican Republi | March to July 2020 | people living in the university | to describe the development, implementation and evaluation of this programme of care in psychological first aid | psychological first aid; psychological first aid, crisis intervention; detection and referral for issues including psychiatric decompensation, violence, suicide attempts, depression crisis, and anxiety crisis; | The UASD COVID-19 Helpline had a team of 62 professionals of psychology divided into three work cohorts, available from Monday to Wednesday, from 7 a.m. to 11 p.m. There were 62 psychologists involved and trained to provide care | **acceptability**: most users reported feeling satisfied; most people indicated they would call the Helpline again if they felt emotionally unwell (96%), and 97% said they would recommend it to other people. | **acceptability**: an assessment survey |
| ***Robotic telemedicine & VR*** | | | | | | | | |
| Gabrielli (2021)[35] | Italy | October to November 2020 | university students | to investigate the potential effect of a healthy-coping chatbot intervention during the COVID-19 pandemic | psychotherapy; CBT, positive psychology, and mindfulness techniques. | The full program consists of eight short sessions, each lasting about 10 minutes, delivered twice a week for 4 weeks. Each session is initiated by the chatbot on a scheduled plan decided by the user during the first session. | **usability**: Participants engaged with the Atena chatbot an average of 78 (SD 24.8) times over the study period; **feasibility**: A total of 61 out of 71 (86%) participants completed the first 2 weeks of the intervention. A total of 41 participants out of 71 (58%) completed the full intervention; **effectiveness**: a significant decrease in anxiety symptoms for participants in more extreme GAD-7 score ranges (P=.009) and a decrease in stress symptoms as measured by the PSS-10 (P=.05) for all participants post-intervention | **usability**: average times of engagement; **feasibility**: adherence; **effectiveness**: PSS-10; GAD-7 |
| Kolbe (2021)[50] | US | late March to late June of 2020 | COVID-19 inpatients | to understand patient satisfaction and perceived benefit of virtual reality on a COVID-19 recovery unit, as well as the logistical and operational feasibility of providing VR content for patients and staff | psychotherapy; meditation, Exploration of natural environments, and Cognitive stimulation games. | VR tool was offered in the inpatient CRU: in the context of guided use with neuropsychologist staff, or with independent use following an introduction to VR. Patients were offered sessions of up to 30 min (actual time with VR headset on was about 10 min). Staff had the option of either experiencing VR mindfulness therapy in a guided format led by a dedicated neuropsychologist, or of self-directing their VR experience during break times at their own discretion. | **acceptability**: high satisfaction across groups; patients’ free-text responses to VR use included ideas of “travel” and “escape; staff emphasized the tool’s utility as a way of enhancing coping and self-care; all patients except for one, and all staff, reported that VR enhanced treatment and well-being, respectively | **acceptability**: a survey on satisfaction and perceived benefit |
| Lima (2021)[52] | India | not specified | people with dementia | to design, develop, and test the feasibility of a social robotic platform to support people with dementia, during and following the COVID-19 pandemic | telemedicine cognitive engagement and mental health support; Affective Hybrid Face Robot; Intelligent Virtual Assistant | a set of three robot-assisted cognitive engagement sessions; presentation of the robot, discussion of newspaper articles, and listening to music | **usability**: an overall positive impression of the multimodal robotic system  **feasibility:** indicates the need to adjust some features | **usability**: user experience questionnaire; **feasibility**: qualitative feedbacks |

*ACT=acceptance and commitment therapy, ASD=autism spectrum disorder, ASDS= Acute Stress Disorder Scale; BAI=Beck Anxiety Inventory, BDI-II=Beck Depression Inventory–II, BSI=Brief Symptom Inventory, CBT=Cognitive Behavioral Therapy,* *CSQ=Client Satisfaction Questionnaire, CTM=clinical team manager, DASS-21=Depression Anxiety Stress Scales-21,* *DBT=dialectical behavior therapy, ED=Eating Disorder,* *EPDS=Edinburgh Postnatal Depression Scale, GAD-7=Generalized Anxiety Disorder-7,* *GDS=Geriatric Depression Scale,* *HADS=Hospital Anxiety and Depression Scale, HAMA=Hamilton Anxiety Rating Scale,* *HAMD=Hamilton Rating Scale for Depression,* *IES-R= Impact of Events Scale—Revised, ISI=Insomnia Severity Index,* *K-10=Ten-Item Kessler Psychological Distress Scale,* *LTEQ= Leisure-Time Exercise Questionnaire, PHP=partial hospital program, PHQ-9=Patient Health Questionnaire-9,* *PPD=Postpartum depression,* *PSS=Perceived Stress Scale, PTSD=Post-Traumatic Stress Disorder,* *RSES=Rosenberg Self-Esteem Scale, SAS=Self-rating Anxiety Scale, SDS=Self-rating Depression Scale, STAI=state-trait anxiety inventory*
