# Supplementary material for: The Usability, Feasibility, Acceptability, and Efficacy of Digital Mental Health Services in the COVID-19 Pandemic: Scoping Review, Systematic Review, and Meta-analysis
Source: JMIR Public Health Surveill. 2023 Feb 13;9:e43730. doi: 10.2196/43730 (PMC9930923; doi:10.2196/43730)
Supplement: Multimedia Appendix 4 [file publichealth_v9i1e43730_app4.docx]

**Multimedia Appendix 4. Results of the meta-regression for** **depression and anxiety.**

**1. Regression of sample size on the effect size of depression**


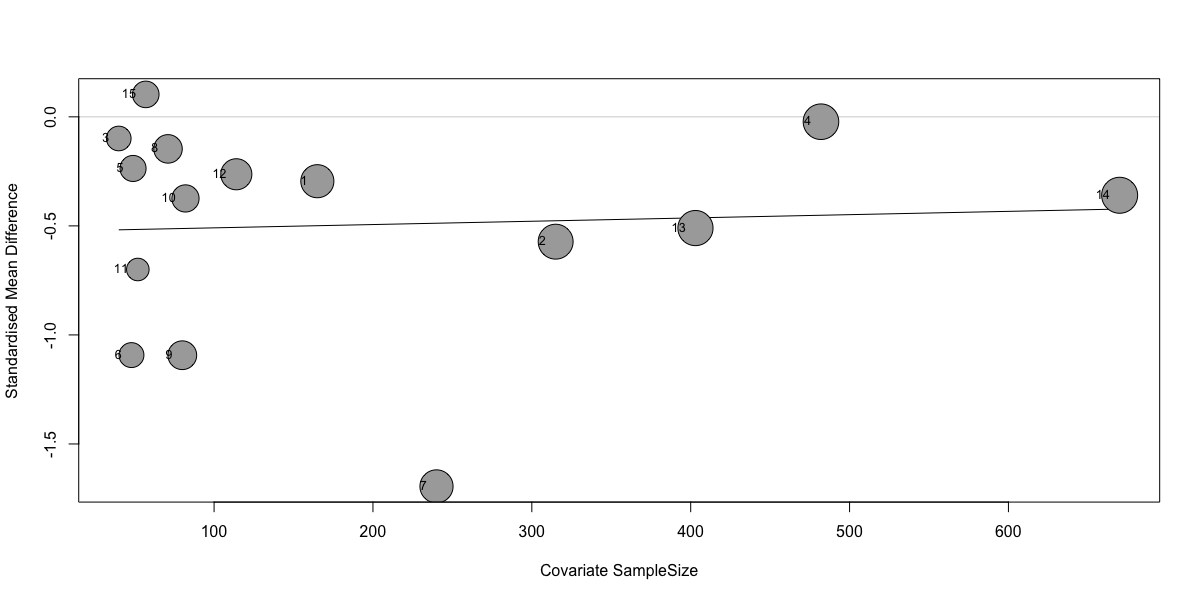


**Figure 1. Meta-regression chart of the effect size of depression by sample size**

Model Results:

estimate se zval pval ci.lb ci.ub

intrcpt -0.5242 0.1956 -2.6803 0.0074 -0.9075 -0.1409 **

SampleSize 0.0002 0.0007 0.2241 0.8227 -0.0012 0.0015

---

Signif. codes: 0 ‘***’ 0.001 ‘**’ 0.01 ‘*’ 0.05 ‘.’ 0.1 ‘ ’ 1

**2. Regression of age on the** **effect size of depression**


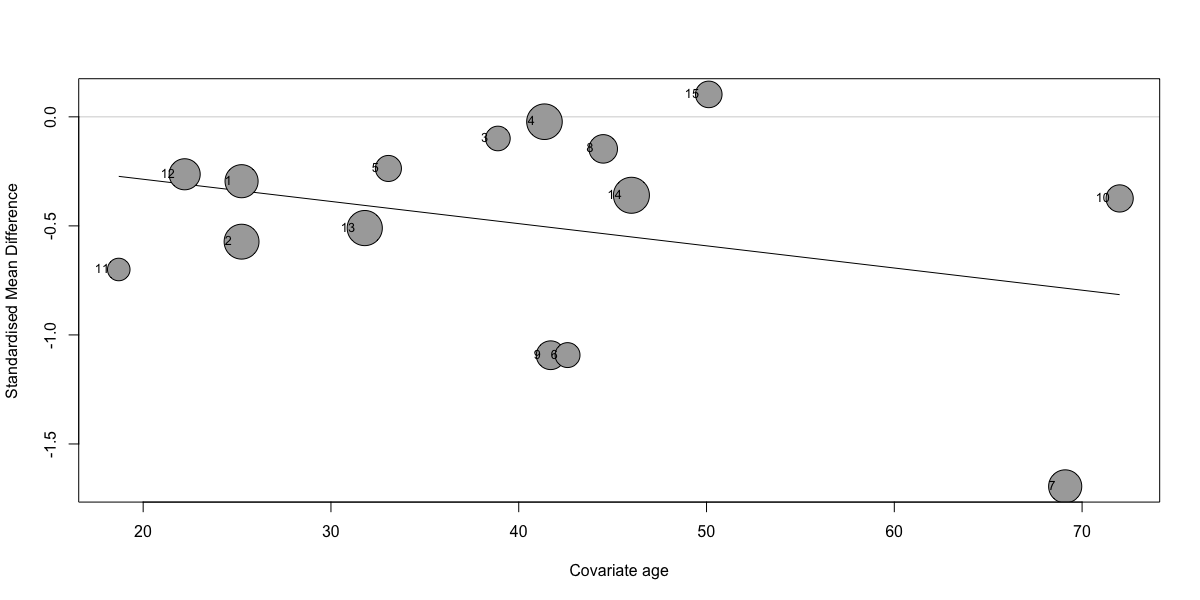


**Figure 2. Meta-regression chart of the effect size of depression by age**

Model Results:

estimate se zval pval ci.lb ci.ub

intrcpt -0.0822 0.3542 -0.2320 0.8165 -0.7764 0.6121

age -0.0102 0.0083 -1.2338 0.2173 -0.0264 0.0060

---

Signif. codes: 0 ‘***’ 0.001 ‘**’ 0.01 ‘*’ 0.05 ‘.’ 0.1 ‘ ’ 1

**3. Regression of gender on the effect size of depression**


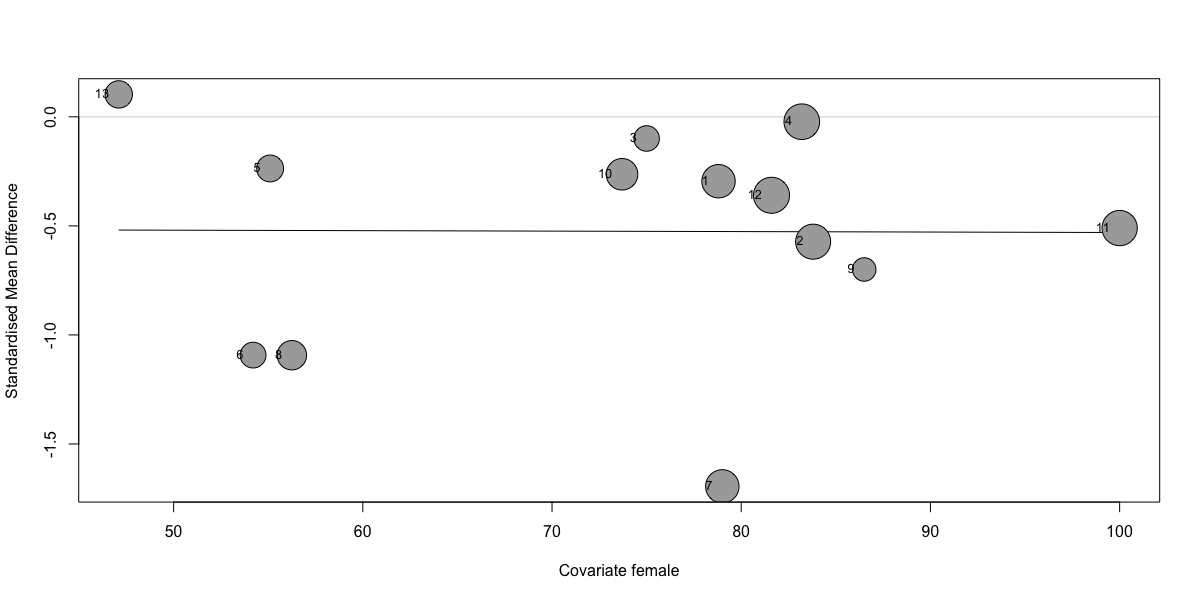


**Figure 3. Meta-regression chart of the effect size of depression by proportion of female**

Model Results:

estimate se zval pval ci.lb ci.ub

intrcpt -0.5085 0.7688 -0.6614 0.5083 -2.0152 0.9983

female -0.0002 0.0101 -0.0224 0.9821 -0.0201 0.0196

---

Signif. codes: 0 ‘***’ 0.001 ‘**’ 0.01 ‘*’ 0.05 ‘.’ 0.1 ‘ ’ 1

**4. Regression of sample size on the effect size of anxiety**


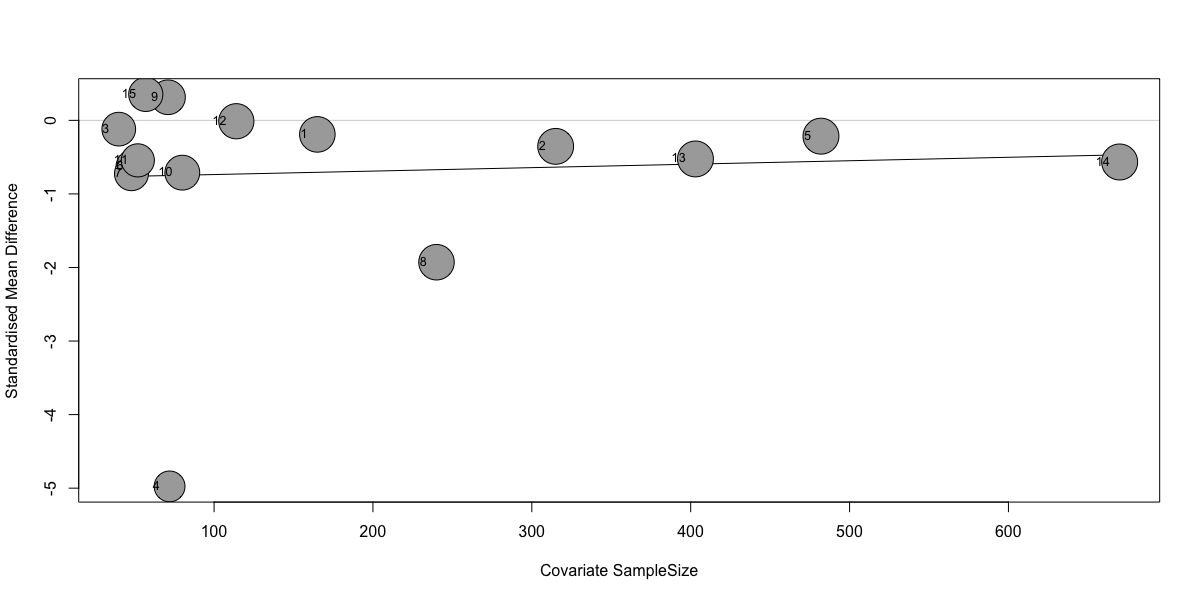


**Figure 4. Meta-regression chart of the effect size of anxiety by sample size**

Model Results:

estimate se zval pval ci.lb ci.ub

intrcpt -0.7835 0.4592 -1.7063 0.0880 -1.6836 0.1165 .

SampleSize 0.0005 0.0017 0.2761 0.7825 -0.0028 0.0038

---

Signif. codes: 0 ‘***’ 0.001 ‘**’ 0.01 ‘*’ 0.05 ‘.’ 0.1 ‘ ’ 1**5. Regression of age on the effect size of anxiety**


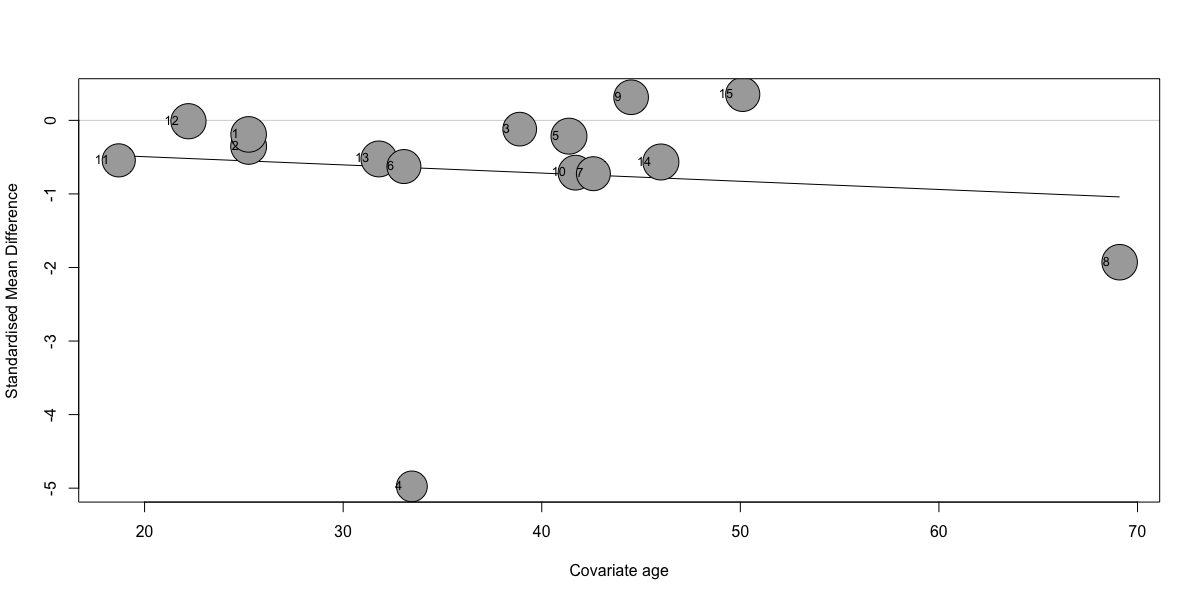


**Figure 5. Meta-regression chart of the effect size of anxiety by age**

Model Results:

estimate se zval pval ci.lb ci.ub

intrcpt -0.2699 1.0107 -0.2670 0.7894 -2.2508 1.7111

age -0.0112 0.0255 -0.4398 0.6601 -0.0611 0.0387

---

Signif. codes: 0 ‘***’ 0.001 ‘**’ 0.01 ‘*’ 0.05 ‘.’ 0.1 ‘ ’ 1

**6. Regression of** **gender on the effect size of anxiety**


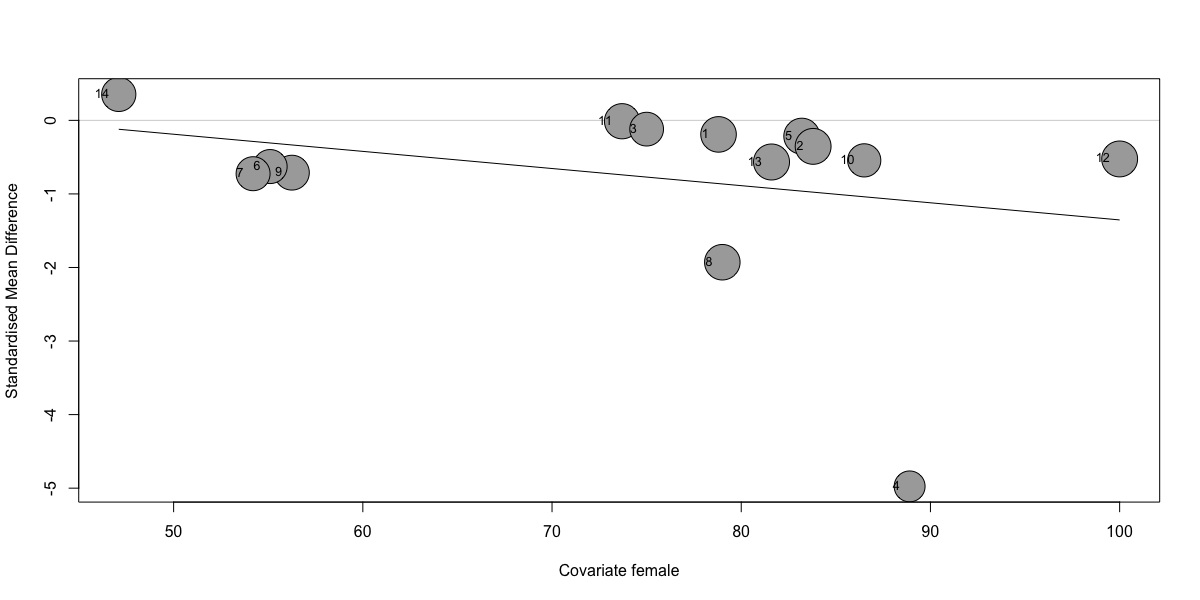


**Figure 6. Meta-regression chart of the effect size of depression by proportion of female**

Model Results:

estimate se zval pval ci.lb ci.ub

intrcpt 0.9787 1.6409 0.5964 0.5509 -2.2375 4.1949

female -0.0234 0.0216 -1.0823 0.2791 -0.0656 0.0189

---

Signif. codes: 0 ‘***’ 0.001 ‘**’ 0.01 ‘*’ 0.05 ‘.’ 0.1 ‘ ’ 1
