# Supplementary material for: The Usability, Feasibility, Acceptability, and Efficacy of Digital Mental Health Services in the COVID-19 Pandemic: Scoping Review, Systematic Review, and Meta-analysis
Source: JMIR Public Health Surveill. 2023 Feb 13;9:e43730. doi: 10.2196/43730 (PMC9930923; doi:10.2196/43730)
Supplement: Multimedia Appendix 5 [file publichealth_v9i1e43730_app5.docx]

**Multimedia Appendix 5. Publication bias of meta-analyses.**

**1. Publication bias of studies evaluated depression**


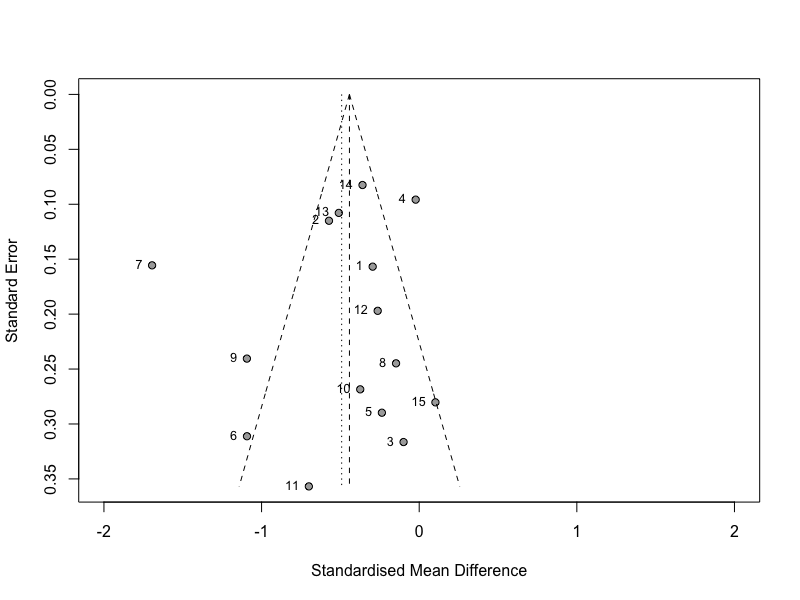


**Figure 7. Funnel plot (Depression)**

**2. Publication bias of studies evaluated anxiety**


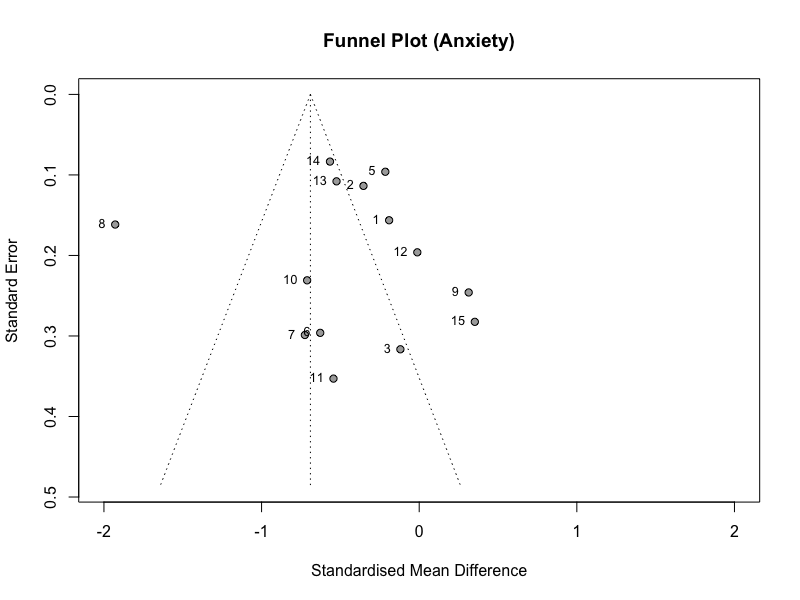


**Figure 8. Funnel plot (Anxiety)**
