# Supplementary material for: The Usability, Feasibility, Acceptability, and Efficacy of Digital Mental Health Services in the COVID-19 Pandemic: Scoping Review, Systematic Review, and Meta-analysis
Source: JMIR Public Health Surveill. 2023 Feb 13;9:e43730. doi: 10.2196/43730 (PMC9930923; doi:10.2196/43730)
Supplement: Multimedia Appendix 6 [file publichealth_v9i1e43730_app6.docx]

**Multimedia Appendix 6. Risk of Bias across randomized studies for meta-analyses.**

**1. ‘Risk of bias’ graph: summary ratings across randomized studies for each risk of bias domain**


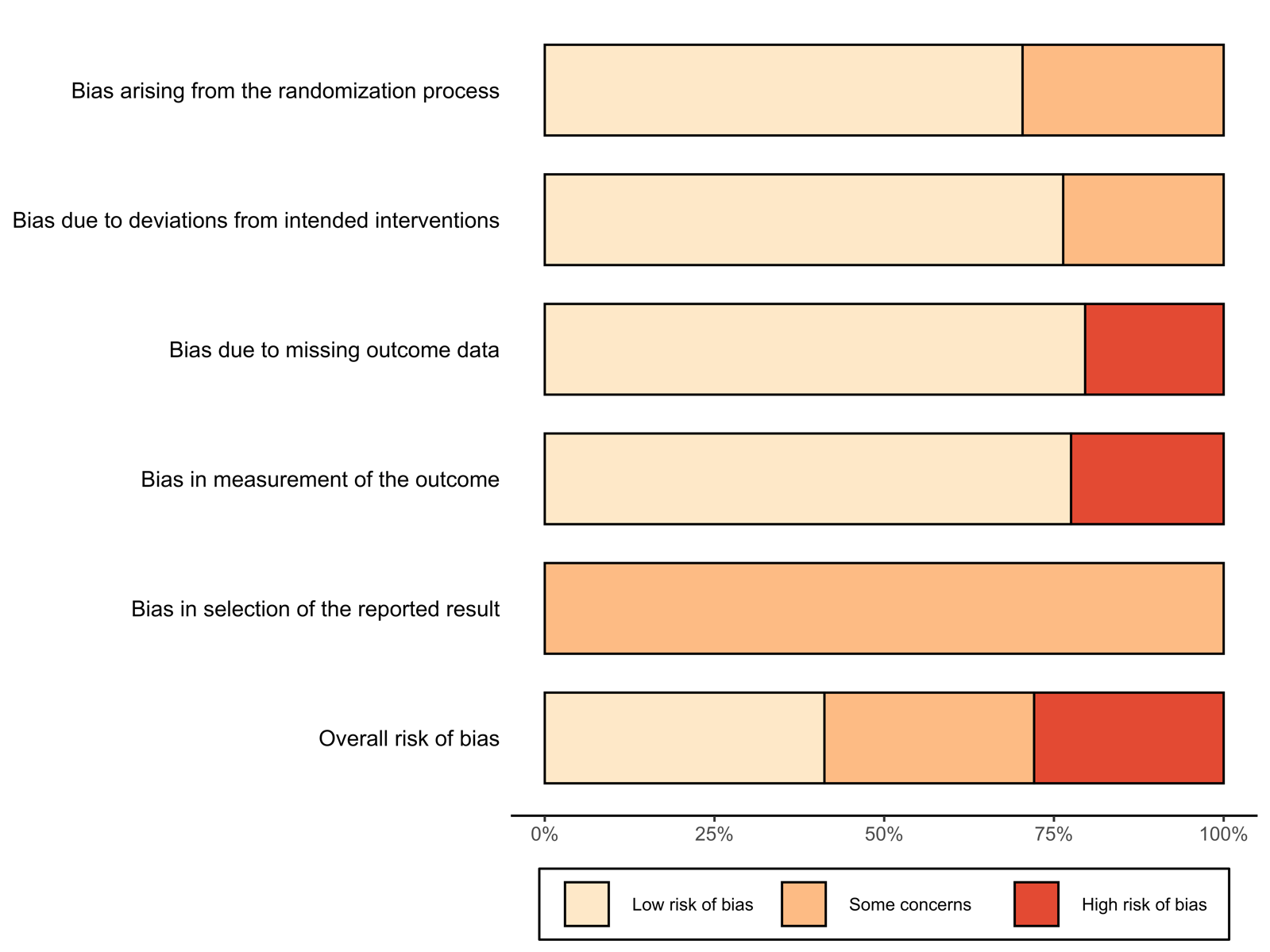


**Figure 9. ‘Risk of bias’ graph: review authors’ ratings across randomized studies for each risk of bias domain**

**2. ‘Risk of bias’ summary: review authors’ ratings about each risk of bias item for each included study.**


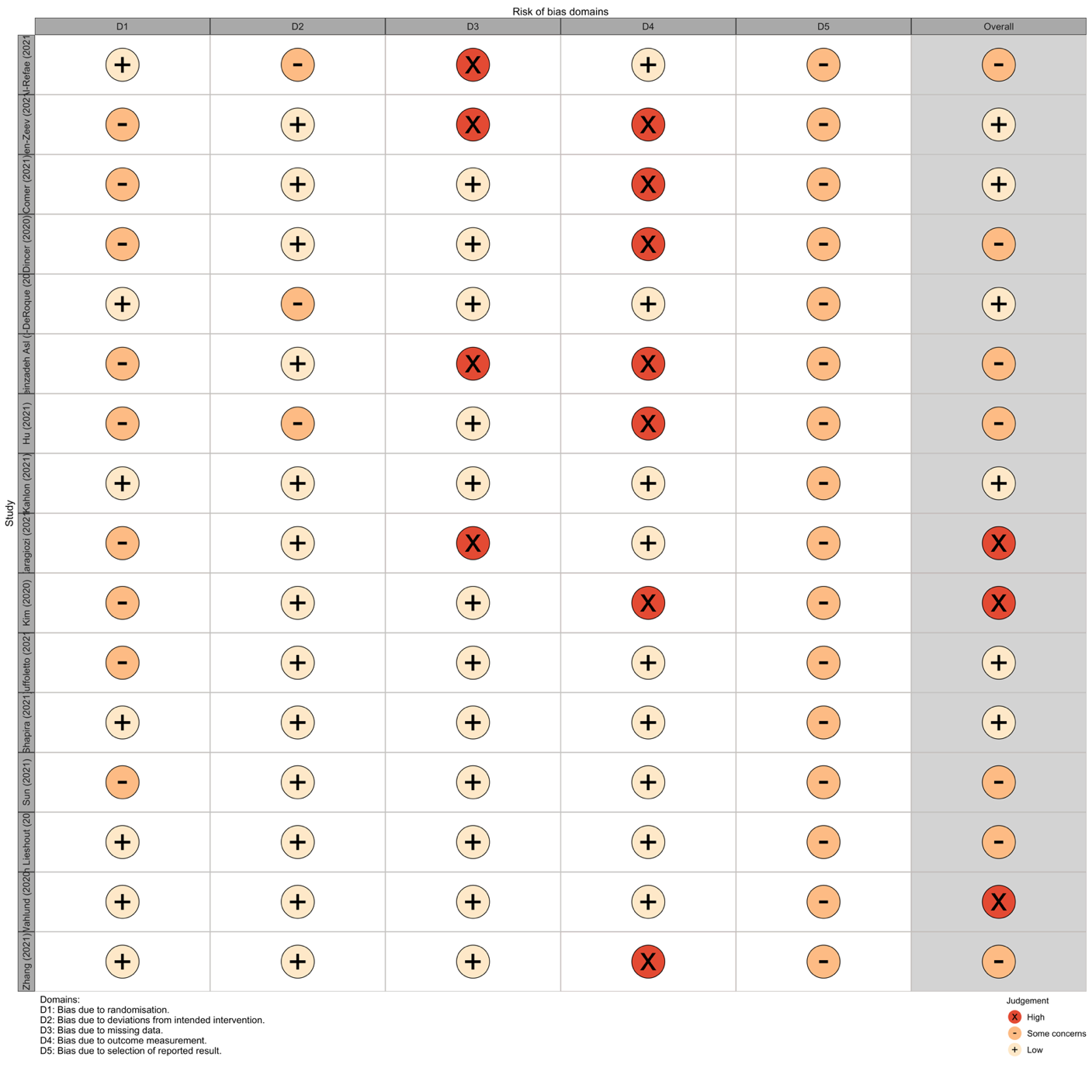


**Figure 10. ‘Risk of bias’** **summary:** **review authors’ ratings about each risk of bias item for each included study.**
